# A signature motif in LIM proteins mediates binding to checkpoint proteins and increases tumour radiosensitivity

**DOI:** 10.1038/ncomms14059

**Published:** 2017-01-17

**Authors:** Xiaojie Xu, Zhongyi Fan, Chaoyang Liang, Ling Li, Lili Wang, Yingchun Liang, Jun Wu, Shaohong Chang, Zhifeng Yan, Zhaohui Lv, Jing Fu, Yang Liu, Shuai Jin, Tao Wang, Tian Hong, Yishan Dong, Lihua Ding, Long Cheng, Rui Liu, Shenbo Fu, Shunchang Jiao, Qinong Ye

**Affiliations:** 1 Department of Medical Molecular Biology, Beijing Institute of Biotechnology, Collaborative Innovation Center for Cancer Medicine, Beijing 100850, China; 2 Institute of Cancer Stem Cell, Cancer Center, Dalian Medical University, Liaoning 116023, China; 3 Department of Oncology, PLA General Hospital, Beijing 100853, China; 4 Department of Thoracic Surgery, Hainan Branch of PLA General Hospital, Hainan 572013, China; 5 Medical Research Center of Shengjing Hospital, China Medical University, Liaoning 110004, China; 6 Department of Microorganism Engineering, Beijing Institute of Biotechnology, Beijing 100071, China; 7 Department of Gynecology and Obstetrics, PLA General Hospital, Beijing 100853, China; 8 Department of Endocrinology, PLA General Hospital, Beijing 100853, China; 9 Department of Thoracic Surgery, PLA General Hospital, Beijing 100853, China; 10 Department of Oncology, 307 Hospital of People's Liberation Army, Beijing 100071, China; 11 Department of Renal Cancer and Melanoma, Peking University Cancer Hospital & Institute, Beijing 100142, China; 12 Department of Radiotherapy, The First Affiliated Hospital of Xi'an Jiao Tong University, Xi'an 710061, China

## Abstract

Tumour radiotherapy resistance involves the cell cycle pathway. CDC25 phosphatases are key cell cycle regulators. However, how CDC25 activity is precisely controlled remains largely unknown. Here, we show that LIM domain-containing proteins, such as FHL1, increase inhibitory CDC25 phosphorylation by forming a complex with CHK2 and CDC25, and sequester CDC25 in the cytoplasm by forming another complex with 14-3-3 and CDC25, resulting in increased radioresistance in cancer cells. FHL1 expression, induced by ionizing irradiation in a SP1- and MLL1-dependent manner, positively correlates with radioresistance in cancer patients. We identify a cell-penetrating 11 amino-acid motif within LIM domains (eLIM) that is sufficient for binding CHK2 and CDC25, reducing the CHK2–CDC25 and CDC25–14-3-3 interaction and enhancing CDC25 activity and cancer radiosensitivity accompanied by mitotic catastrophe and apoptosis. Our results provide novel insight into molecular mechanisms underlying CDC25 activity regulation. LIM protein inhibition or use of eLIM may be new strategies for improving tumour radiosensitivity.

## Abstract (web summary)

CDC25 phosphatases are important cell cycle regulators. Here, the authors show that the LIM domain-containing proteins (for example, FHL1) induce inhibitory CDC25 phosphorylation resulting in radioresistance and that a specific peptide can increase tumour radiosensitivity by increasing CDC25 activity.

Mammalian cells respond to DNA-damaging agents, such as ionizing radiation (IR), by activating cell cycle checkpoints^1^,^2^. Cell cycle checkpoints are control mechanisms that ensure genome stability. Defects in the checkpoints have been exploited therapeutically in the treatment of cancer with radiotherapy^3^,^4^,^5^. However, radioresistance is a major obstacle to radiotherapy for many cancers. Thus, elucidating molecular mechanisms underlying cell cycle checkpoints may provide new therapeutic targets for overcoming radioresistance. Cell division cycle 25 (CDC25) family phosphatases, consisting of CDC25A, B and C, play key roles in regulation of cell cycle progression under normal conditions and after DNA damage^6^,^7^. CDC25C has been extensively characterized in terms of function and regulation since its discovery as the first Cdc25 phosphatase. When phosphorylated at serine 216 (S216) by the checkpoint kinases CHK1 and CHK2 in response to DNA damage, human CDC25C binds to members of the 14-3-3 family of proteins, sequestering CDC25C in the cytoplasm and thereby inhibiting CDC25C phosphatase activity^8^,^9^. Negative regulation of CDC25C activity by phosphorylation on S216 and cytoplasmic sequestration is an important regulatory mechanism used by cells to block mitotic entry and repair damaged DNA. However, how CDC25 activity is precisely negatively regulated remains largely unknown.

LIM (Lin11, Isl-1 and Mec-3) domains are cysteine-rich zinc finger motifs mediating protein–protein interactions with transcription factors, cell-signalling molecules and cytoskeleton-associated proteins^10^,^11^,^12^. The consensus amino-acid sequence for the LIM domain based on 135 human LIM sequences is CX_2_CX_16–23_C/HX_2/4_C/H/EX_2_CX_2_CX_14–21_C/HX_1/2/3_C/H/D/EX (with X any amino acid). Through interaction with cellular proteins, LIM domain-containing proteins (LIM proteins) regulate many cellular processes. We and others have shown that expression of four-and-a-half LIM domain protein 1 (FHL1), characterized by four complete LIM domains preceded by an N-terminal half LIM domain, is downregulated in many cancers^13^,^14^,^15^,^16^,^17^,^18^,^19^, such as breast cancer, prostate cancer, liver cancer, lung cancer and gastric cancer. FHL1 inhibits cancer cell growth, migration and invasion.

To understand the mechanisms underlying the role of FHL1 in cancer, we have performed yeast two-hybrid screens using FHL1 as bait^20^, and identified CHK2, CDC25C and 14-3-3ɛ as novel FHL1-interacting proteins. Since CHK2, CDC25C and 14-3-3 proteins are key components of the G2/M cell cycle checkpoint network in response to IR^21^,^22^,^23^, we investigated the role of LIM proteins (FHL1-3, LMO1-4 and CRP), with FHL1 as a representative LIM protein, in cell cycle checkpoint regulation. We found that IR-inducible LIM proteins, such as FHL1, inhibit CDC25C activity, resulting in radioresistance in cancer cells. We have further identified eleven amino acids within the LIM domain (eLIM) as a signature motif mediating binding to CHK2 and CDC25C. Importantly, eLIM peptides are cell-permeable and increases radiation sensitivity in cancer cells by increasing CDC25 activity.

## Results

### LIM proteins interact with CHK2/CDC25/14-3-3

**Figure 1:**
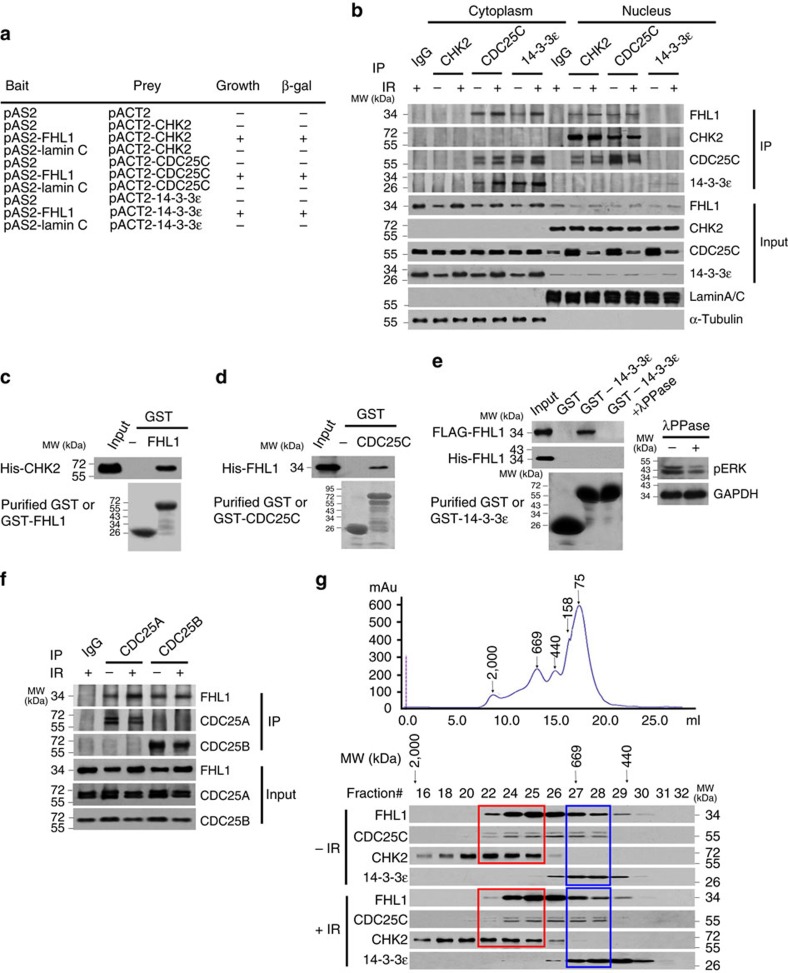
FHL1 interacts with CHK2/CDC25C/14-3-3ɛ. (**a**) Yeast CG1945 cells were transformed with the indicated plasmids (bait and prey for the two-hybrid assay) and grown on selective media. Positive interaction is indicative of colonies that grow on selective media and have β-galactosidase activity. (**b**) HeLa cells were treated with or without IR (8 Gy). Six hours later, cells were fractionated into cytoplasmic and nuclear fractions, and IP with the indicated antibodies or preimmune control serum (IgG). Precipitates were analysed by immunoblot using the indicated antibodies. Lamin A/C and α-tubulin were used as the nuclear and cytoplasmic marker, respectively. MW, molecular weight. (**c**,**d**) Glutathione-sepharose beads bound with GST-FHL1 (**c**), GST-CDC25C (**d**) or GST were incubated with purified His-tagged CHK2 (**c**) or FHL1 (**d**). After washing the beads, the bound proteins were subjected to SDS–PAGE and immunoblot with anti-His antibody. (**e**) Glutathione-sepharose beads bound with GST-14-4-3ɛ or GST were incubated with FLAG-FHL1-expressing HeLa cell lysates or purified His-tagged-FHL1 treated with or without the protein phosphatase λPPase. The bound proteins were analysed by immunoblot with anti-FLAG or anti-His. As a positive control for λPPase, HeLa whole cell lysates were treated with λPPase, and the treated lysates showed decreased ERK1/2 phosphorylation (pERK1/2) with immunoblot. (**f**) HeLa cell lysates were IP with antibodies specific for CDC25A or CDC25B, followed by immunoblotting with the indicated antibodies. (**g**) HeLa cell extracts were fractionated on Superose 6 size exclusion columns. FPLC chromatographic elution profiles are shown. The elution positions of calibration proteins with known molecular masses (kDa) are indicated by arrows, and an equal volume from each chromatographic fraction was analysed by immunoblot with the indicated antibodies. Red frame indicates a potential complex with FHL1, CDC25C and CHK2, and blue frame shows a potential complex with FHL1, CDC25C and 14-3-3ɛ.

Using FHL1 as a bait in the yeast two-hybrid system^20^, we identified the G2/M checkpoint-related proteins CHK2, CDC25C and 14-3-3ɛ, which bound FHL1 (Fig. 1a). Endogenous FHL1 interacted with endogenous CDC25C, from both cytoplasmic and nuclear fractions of cervical cancer HeLa cells, in the absence or presence of IR (Fig. 1b). Endogenous FHL1 associated with endogenous CHK2 in the nucleus, whereas it associated with endogenous 14-3-3ɛ in the cytoplasm (Fig. 1b). Intriguingly, like 14-3-3ɛ, FHL1 was IR-inducible. CDC25C protein levels were decreased after IR as previously reported^2^. The interaction between FHL1 and CHK2 or CDC25C is direct, because purified His-tagged CHK2 protein interacted with purified GST-FHL1 protein, but not GST alone (Fig. 1c), and purified His-tagged-FHL1 protein bound to purified CDC25C (Fig. 1d). Treatment of FLAG-FHL1-overexpressing cell lysate with the protein phosphatase λPPase completely abolished the ability of FHL1 to interact with 14-3-3ɛ, and purified His-tagged-FHL1 did not interact with purified 14-3-3ɛ, suggesting that phosphorylated FHL1 associates with 14-3-3ɛ (Fig. 1e). FHL1 did not interact with the CDC25C substrate CDC2 (Supplementary Fig. 1a) and CHK1 (Supplementary Fig. 1b). Besides CDC25C, FHL1 also bound to CDC25A and CDC25B (Fig. 1f). 14-3-3ɛ, 14-3-3σ and 14-3-3ζ were shown to bind to CDC25 (refs 24, 25, 26). FHL1 interacted with 14-3-3ɛ and 14-3-3σ, but not 14-3-3ζ (Supplementary Fig. 1c). Protein fractionation experiments performed by fast protein liquid chromatography (FPLC) revealed that, in the presence or absence of IR, the elution pattern of FHL1 overlapped with that of the CDC25C and CHK2 proteins or that of the CDC25C and 14-3-3ɛ proteins (Fig. 1g). The reason why there are no marked protein mobility differences between IR and non-IR samples may be that the protein complexes are so large (>440 kDa) that posttranslational modifications of the components of the complexes, such as CDC25C and CHK2, may not be sufficient for alteration of the size of the protein complexes due to limited resolution of the FPLC used. These results, combined with the data shown above and below, suggest that FHL1 forms one complex with CDC25C and CHK2, and forms another complex with CDC25C and 14-3-3ɛ.

To test whether other LIM proteins interact with CHK2, CDC25C and 14-3-3ɛ, we selected FHL2, FHL3, LMO1-4 and CRP. Interestingly, except CRP and LMO4, all these LIM proteins interacted with CHK2, CDC25C and 14-3-3ɛ (Supplementary Fig. 1b,d–f).

Domain mapping showed that FHL1 specifically bound to CHK2 (220–356) containing the N-terminal portion of the protein kinase domain (Supplementary Fig. 2a). FHL1 did not interact with CDC25C (Δ328–383), a naturally occurring CDC25C isoform we obtained from a human mammary cDNA library, suggesting that the region between amino acid (aa) 328 and 383 of CDC25C containing partial catalytic domain contributes to the FHL1–CDC25C interaction (Supplementary Fig. 2b). FHL1 specifically interacted with 14-3-3ɛ (100–255) containing the target binding pocket (Supplementary Fig. 2c).

### LIM proteins regulate CDC25 phosphorylation by CHK2

**Figure 2:**
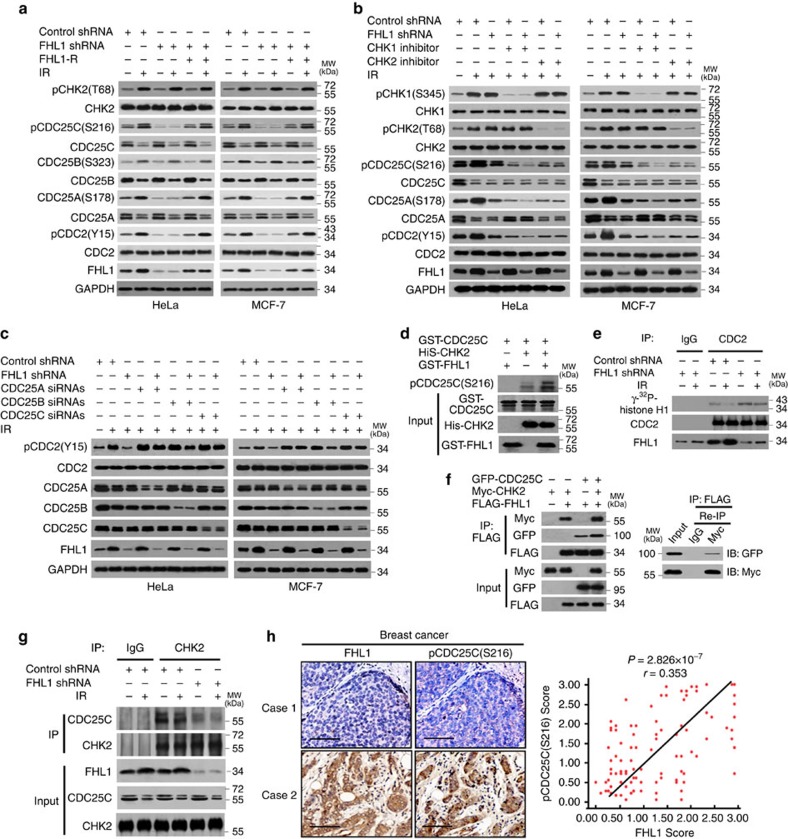
FHL1 regulates CDC25 activity via CHK2-mediated CDC25 phosphorylation. (**a**) Immunoblot analysis of HeLa or MCF-7 cells stably infected with lentivirus carrying FHL1 shRNA or FHL1 shRNA plus siRNA-resistant FHL1 (FHL1-R), exposed to IR (8 Gy) and harvested 6 h after IR. GAPDH was used as a loading control. (**b**) Immunoblot analysis of HeLa or MCF-7 cells stably infected with FHL1 shRNA or control shRNA, treated with 50 nM CHK1 inhibitor SB218078 (Tocris) or 100 nM CHK2 inhibitor II (Sigma-Aldrich) and exposed to IR (8 Gy). Cells were harvested 6 h after IR and analysed with the indicated antibodies. (**c**) HeLa or MCF-7 cells stably infected with FHL1 shRNA or control shRNA were transfected with siRNAs for CDC25A, CDC25B or CDC25C for 48 h. Cells were treated with IR and analysed by immunoblot with the indicated antibodies. (**d**) *In vitro* kinase assay of purified His-tagged CHK2 incubated with purified GST-CDC25C or GST-CDC25C plus purified GST-FHL1. Immunoblot was performed with the indicated antibodies. (**e**) HeLa cells stably infected with FHL1 shRNA or control shRNA were irradiated and IP with anti-CDC2 or normal IgG. *In vitro* kinase assays were performed using the immunoprecipitates and the substrate GST-histone H1, and analysed by SDS–PAGE and immunoblot or autoradiography as indicated. (**f**) HeLa cells transfected with the indicated plasmids were IP with anti-FLAG. The immune complexes were eluted with FLAG peptide and Re-IP using anti-Myc or normal IgG. The resulting precipitates were analysed by immunoblotting with the indicated antibodies. (**g**) HeLa cells stably infected with FHL1 shRNA or control shRNA were irradiated and IP with anti-CHK2 or normal IgG. The precipitates were analysed by immunoblotting with the indicated antibodies. (**h**) Representative immunohistochemical staining of FHL1 and pCDC25C (S216) in 104 human breast cancer samples. Case 1 and case 2 refer to two representative samples categorized by low and high expression of FHL1 and pCDC25C (S216). Scale bars, 100 μm. The correlation of FHL1 with pCDC25C (S216) is shown. The *P* value was generated using the Spearman's rank correlation test.

In response to IR, human CHK2 is activated by increased phosphorylation of threonine 68 (T68)^8^,^9^. Activated CHK2 phosphorylates CDC25C on S216, a site known to be involved in 14-3-3 binding and negative regulation of CDC25C. CDC25C can dephosphorylate CDC2 at tyrosine 15 (Y15), promoting entry into mitosis^8^,^9^. Thus, negative regulation of CDC25C by CHK2 results in increased phosphorylation of CDC2 (Y15). We investigated whether FHL1 regulates the CHK2–CDC25–CDC2 pathway. FHL1 knockdown in human cervical cancer HeLa and breast cancer MCF-7 cells reduced phosphorylation of CDC25C (S216) and CDC2 (Y15) (Fig. 2a and Supplementary Fig. 3a). Compared with normal conditions, IR had a more marked effect. Phosphorylation of CDC25A on S178 and CDC25B on S323 is involved in 14-3-3 binding^27^,^28^. FHL1 knockdown reduced phosphorylation of CDC25A (S178), but not CDC25B (S323) (Fig. 2a). The knockdown effects could be rescued by siRNA-resistant FHL1 expression. Similar results were obtained with mouse embryonic fibroblasts (MEFs) from FHL1 knockout (KO) mice (Supplementary Fig. 3b,c) except that CDC25C (S216) phosphorylation could not be detected in MEFs because mouse CDC25C does not have an equivalent to the S216 phosphorylation site^29^. In addition, mouse CDC25C only had one band shown by immunoblot. Like FHL1 knockdown, FHL2 or FHL3 knockdown also inhibited phosphorylation of CDC25C (S216) and CDC2 (Y15) (Supplementary Fig. 3a,d). Except overexpression of CRP and LMO4, overexpression of all the LIM proteins tested increased phosphorylation of CDC25C (S216) and CDC2 (Y15) (Supplementary Fig. 3e), suggesting that many LIM proteins regulate CDC25C activity. Activation of CHK2, but not CHK1, is required for FHL1 modulation of CDC25C (S216), CDC25A (S178) and CDC2 (Y15) phosphorylation, because the CHK2 inhibitor, which reduces CHK2 (T68) phosphorylation, but not the CHK1 inhibitor, which reduces CHK1 (S345) phosphorylation, abolished the ability of FHL1 knockdown or KO to regulate these phosphorylation events (Fig. 2b and Supplementary Fig. 3f). Furthermore, knockdown of CDC25A/C, but not CDC25B, greatly reduced the ability of FHL1 to regulate CDC2 (Y15) phosphorylation (Fig. 2c). Consistent with the previously reported results showing that *CDC25C* KO mice has a normal checkpoint response to DNA damage possibly due to compensatory mechanisms^29^, *CDC25C* KO MEFs had little effect on CDC2 (Y15) phosphorylation (Supplementary Fig. 3g). However, *CDC25C* KO greatly attenuated the ability of FHL1 to regulate CDC2 (Y15) phosphorylation, suggesting that mouse CDC25C, possibly other phosphorylation sites of mouse CDC25C different from human CDC25C (S216), is involved in FHL1-medaited modulation of CDC2 (Y15) phosphorylation in MEFs.

To test whether FHL1 directly regulates CDC25C (S216) phosphorylation, we performed in *vitro* kinase assay. Although FHL1 failed to directly phosphorylate CDC25C, FHL1 increased CHK2-mediated phosphorylation of CDC25C (S216) (Fig. 2d). In addition, FHL1 knockdown cancer cells or FHL1 KO MEFs had enhanced CDC2 activity (Fig. 2e and Supplementary Fig. 3h). FHL1, CHK2 and CDC25C formed a complex in HeLa cells (Fig. 2f), and FHL1 knockdown or KO greatly blocked the interaction between CHK2 and CDC25C (Fig. 2g and Supplementary Fig. 3i). Importantly, FHL1 expression positively correlated with the phosphorylation level of CDC25C (S216) in breast cancer patients (Fig. 2h). Those with tumours that highly expressed pCDC25C (S216) revealed significantly poorer disease-free survival (DFS), but not overall survival (OS), than those with tumours with low pCDC25C (S216) expression (*P*=0.033) (Supplementary Fig. 4a). There were no significant differences in both DFS and OS regardless of the FHL1 amounts in the tumours (Supplementary Fig. 4b). We confirmed the specificity of the FHL1 and pCDC25C (S216) antibodies by immunohistochemistry (IHC) with corresponding blocking proteins and immunoblot with relative proteins (Supplementary Fig. 4c–f). Taken together, these data suggest that FHL1 plays an important role in regulation of the CHK2–CDC25–CDC2 pathway.

### LIM proteins sequester CDC25C in the cytoplasm via 14-3-3

**Figure 3:**
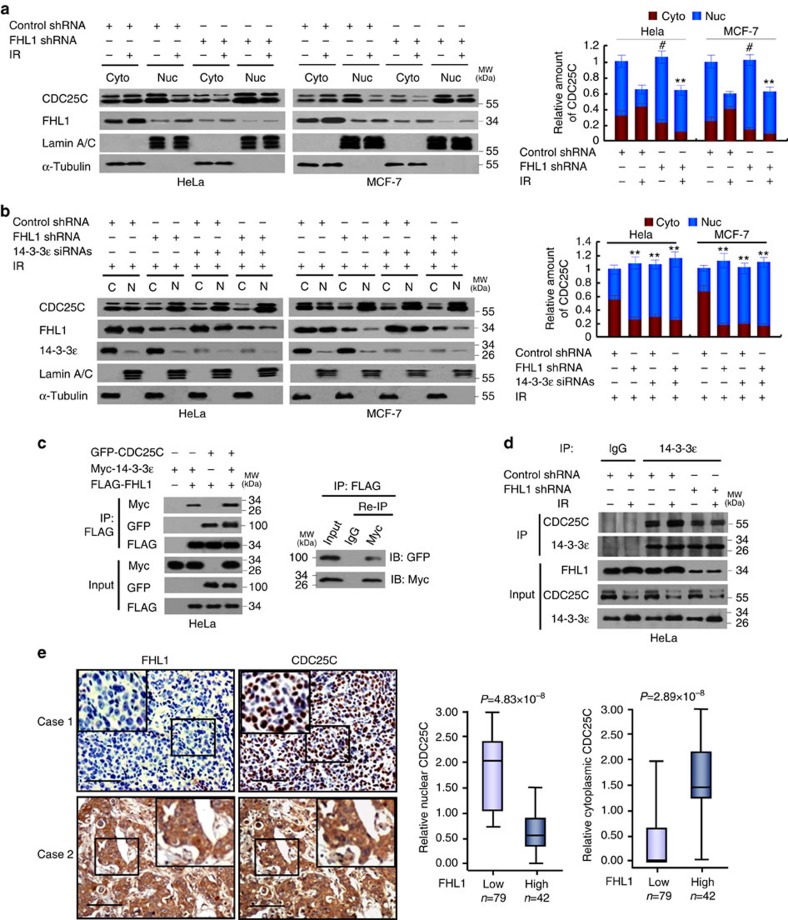
FHL1 sequesters CDC25C in the cytoplasm via 14-3-3ɛ. (**a**) HeLa or MCF-7 cells stably infected with FHL1 shRNA or control shRNA were exposed to IR, fractionated into cytoplasmic (Cyto) and nuclear (Nuc) fractions and analysed by immunoblot with the indicated antibodies. (**b**) HeLa or MCF-7 cells stably infected with FHL1 shRNA or control shRNA were transfected with 14-3-3ɛ siRNAs, exposed to IR, fractionated into cytoplasmic and nuclear fractions, and analysed by immunoblot with the indicated antibodies. C, cytoplasm; N, nucleus. Right panels show the densitometric quantitation of cytoplasmic and nuclear distribution of CDC25C (**a**,**b**). Values shown are mean±s.d. of three independent experiments. The *P* values were generated using two-tailed Student's *t*-test. ^#^*P*<0.05 versus control shRNA without IR (the ratio of nucleus to cytoplasm), **P<0.01 versus control shRNA with IR (**a**,**b**). (**c**) HeLa cells transfected with the indicated plasmids were IP with anti-FLAG. The immune complexes were eluted with FLAG peptide, Re-IP using anti-Myc and analysed by immunoblotting with the indicated antibodies. (**d**) HeLa cells stably infected with FHL1 shRNA or control shRNA were irradiated and IP with anti-14-3-3ɛ or normal IgG. The resulting precipitates were analysed by immunoblotting with the indicated antibodies. (**e**) Representative immunohistochemical staining of FHL1 and CDC25C in human breast cancer samples. Case 1 and case 2 refer to two representative samples categorized by low and high expression of FHL1 and nuclear and cytoplasmic expression of CDC25C. Scale bars, 100 μm. The correlation of FHL1 with nuclear or cytoplasmic CDC25C is shown. The correlation of FHL1 with nuclear or cytoplasmic CDC25C was determined using the Mann–Whitney *U* test. Nuclear and cytoplasmic expression scores are shown in box plots, with the horizontal lines representing the median; the bottom and top of the boxes representing the 25th and 75th confidence limits; and the vertical bars representing the range of data.

When phosphorylated at S216, CDC25C binds to 14-3-3 proteins, sequestering CDC25C in the cytoplasm and thus preventing premature mitosis^8^,^9^. Since FHL1 increases CDC25C (S216) phosphorylation and interacts with 14-3-3, we first determined whether FHL1 regulates CDC25C localization. FHL1 knockdown in HeLa and MCF-7 cells or FHL1 KO in MEFs increased nuclear accumulation of CDC25C with or without IR (Fig. 3a and Supplementary Fig. 5a,b). IR had a more marked effect. Similar results were observed with FHL2 or FHL3 knockdown (Supplementary Fig. 5c). Except overexpression of CRP and LMO4, overexpression of all the LIM proteins tested sequestered CDC25C in the cytoplasm (Supplementary Fig. 5d), indicating that many LIM proteins regulate CDC25C localization. Knockdown of 14-3-3ɛ almost abolished the ability of FHL1 to modulate CDC25C localization (Fig. 3b and Supplementary Fig. 6a). Consistent with this, FHL1 formed a complex with CDC25C and 14-3-3ɛ in HeLa cells (Fig. 3c), and FHL1 knockdown or KO markedly inhibited the CDC25C–14-3-3ɛ interaction (Fig. 3d and Supplementary Fig. 6b). Moreover, in breast cancer patients, high FHL1 expression positively correlated with cytoplasmic CDC25C accumulation and negatively correlated with nuclear CDC25C accumulation (Fig. 3e). There were no significant differences in DFS and OS regardless of the cytoplasmic or nuclear CDC25C amounts (Supplementary Fig. 7a). We confirmed the specificity of the CDC25C antibody by IHC with corresponding blocking proteins and immunoblot using CDC25C knockdown cell lysates (Supplementary Fig. 7b,c). These data suggest that LIM proteins such as FHL1 sequester CDC25C in the cytoplasm via 14-3-3.

### SP1- and MLL1-dependent LIM protein induction by IR

FHL2 was shown to be IR-inducible although the molecular mechanism is unclear^30^. We tested whether other LIM proteins are also IR-inducible. Indeed, FHL1-3 proteins are induced by IR in a dose-dependent manner (Supplementary Fig. 8a). *FHL1-3* and *LMO1-3*, but not *LMO4* and *CRP*, were induced by IR at the transcription level (Supplementary Fig. 8b,c).

To investigate the mechanism underlying the IR-inducible *FHL1* expression, we investigated induction of the *FHL1* promoter after IR. Analysis of various *FHL1* promoter deletion reporter constructs showed that the promoter region from −567 to −548 bp contained an IR-responsive element, whose inducibility was comparable to that of early growth response 1 (Egr1)^31^, a previously reported IR-inducible gene (Supplementary Fig. 8d). Mutation of a putative specific protein 1 (SP1)-binding site in this region predicted by a bioinformatics method (http://tfbind.hgc.jp) resulted in loss of the inducibilty. Consistent with this, ChIP assay showed that SP1 was recruited to the *FHL1* promoter but not to a region approximately 2-kb upstream of the *FHL1* promoter (Supplementary Fig. 8e). Moreover, mono- and dimethylation of histone H3 at lysine 4 (H3K4me and H3K4me2), active marks for transcription and the H3K4 methytransferase MLL1 were enriched at the *FHL1* promoter containing SP1-binding site (Supplementary Fig. 8e). IR treatment further increased the enrichment. Although H3K4me3 and the H3K4 methytransferases SET1A and SETD7 were enriched at the previously reported promoters^32^,^33^,^34^, they were not enriched at the *FHL1* promoter. Re-ChIP experiments showed that SP1 associated with MLL1 on the SP1-binding site of *FHL1* promoter (Supplementary Fig. 8f). SP1 physically interacted with MLL1 and the interaction was increased after IR treatment (Supplementary Fig. 8g). The interaction of SP1 with MLL1 was unlikely to be mediated by chromatin, as it was not affected by the treatment with DNase I that nonspecifically cleaves DNA. Moreover, knockdown of SP1 or MLL1 abrogated the induction of FHL1 after IR (Supplementary Fig. 9), indicating SP1- and MLL1-dependent FHL1 induction. Intriguingly, SP1 knockdown also abolished the IR induction of MLL1.

### LIM proteins regulate G2/M checkpoint and radiosensitivity

**Figure 4:**
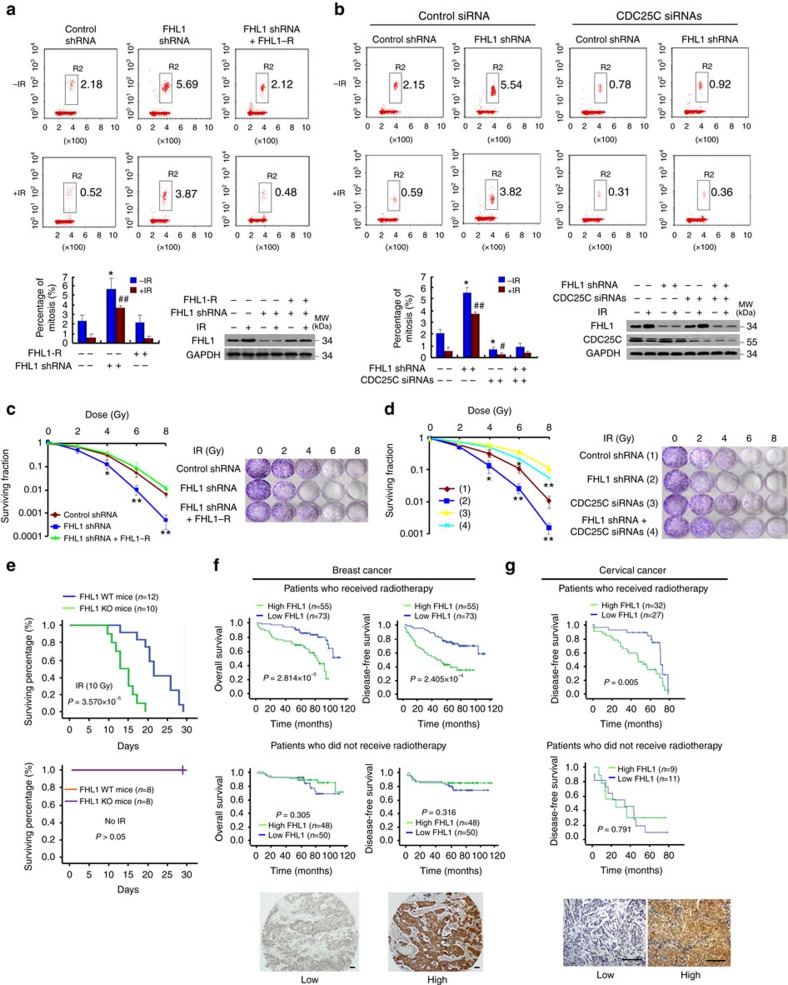
FHL1 regulates cancer radioresistance and is a radioresistance marker. (**a**) HeLa cells infected with FHL1 shRNA or FHL1 shRNA plus FHL1-R were irradiated (8 Gy) and analysed for the proportion of mitotic cells by FACS using phosphorylated histone H3 (Ser 10)-PI staining. Phospho-histone H3 (Ser10) is the mitosis marker. Representative FACS and immunoblot with the indicated antibodies are shown. (**b**) HeLa cells stably infected with FHL1 shRNA or control shRNA were transfected with CDC25C siRNAs, irradiated and analysed as in **a**. Data shown are mean±s.d. of three independent experiments. *P<0.05 versus control shRNA without IR, ^#^*P*<0.05, ^##^*P*<0.01 versus control shRNA with IR (**a**,**b**). (**c**) Clonogenic survival assays of HeLa cells infected with FHL1 shRNA or FHL1 shRNA plus FHL1-R and irradiated at the indicated doses. (**d**) Clonogenic survival assays of FHL1 shRNA-expressing HeLa cells transfected with CDC25C siRNAs and irradiated at the indicated doses. Data shown are mean±s.d. of three independent experiments. *P<0.05, **P<0.01 versus corresponding control shRNA (**c**,**d**). The *P* values were generated using two-tailed Student's *t*-test (**b**–**d**). (**e**) Kaplan–Meier estimates of radiosensitivity of *FHL1* KO mice treated with or without IR (10 Gy). (**f**) Kaplan–Meier estimates of DFS and OS of breast cancer patients with (upper panel) or without (lower panel) radiotherapy. (**g**) Kaplan–Meier estimates of DFS of cervical cancer patients with (upper panel) or without (lower panel) radiotherapy. Marks on graph lines represent censored samples (**f**,**g**). Representative immunohistochemical staining of FHL1 is shown at the bottom (**f**,**g**). Scale bars, 100 μm.

Since LIM proteins regulate CDC25 activity, we tested whether LIM proteins can modulate the G2/M checkpoint. FHL1 knockdown in cancer cells or *FHL1* KO in MEFs abrogated IR-induced G2/M arrest (Fig. 4a and Supplementary Fig. 10a). In the absence of IR, FHL1 had relatively less effect. The knockdown or KO effects could be rescued by FHL1 re-expression. Knockdown of FHL2 or FHL3 had similar effects to FHL1 knockdown on G2/M checkpoint control (Supplementary Fig. 10b,c). Moreover, overexpression of FHL1-3 and LMO1-3, but not LMO4 and CRP, increased IR-induced G2/M arrest (Supplementary Fig. 10d). FHL1 regulated G2/M arrest mainly through CDC25C because CDC25C knockdown in cancer cells or *CDC25C* KO in MEFs greatly abolished such effect (Fig. 4b and Supplementary Fig. 11a).

We then investigated the effects of FHL1 on the viability of cultured cells treated with IR. FHL1 knockdown cancer cells or *FHL1* KO MEFs exhibited decreased survival on exposure to IR (Fig. 4c and Supplementary Fig. 11b). The knockdown effects could be rescued by the expression of siRNA-resistant FHL1. CDC25C knockdown in cancer cells or CDC25C KO in MEFs greatly abrogated the ability of FHL1 knockdown by increasing the survival of the irradiated cells (Fig. 4d and Supplementary Fig. 11c). Like FHL1 knockdown, FHL2 or FHL3 knockdown showed decreased survival (Supplementary Fig. 12a,b). In addition, overexpression of FHL1-3 and LMO1-3, but not LMO4 and CRP, revealed increased survival of the irradiated cells (Supplementary Fig. 12c,d). Elevated levels of FHL1-3 were observed in radiation-resistant oesophageal carcinoma Eca109 cell line as compared with the parental cell lines (Supplementary Fig. 12e). FHL1, FHL2 or FHL3 knockdown sensitizes the radiation-resistant cells to IR, accompanied by decreased phosphorylation of CDC25C (S216) and CDC2 (Y15). Furthermore, compared with HeLa cells, *FHL1* or *14-3-3ɛ* KO HeLa cells generated by CRISPER/Cas9 were more sensitive to IR, while *CDC25C* KO HeLa cells were more resistant to IR (Supplementary Fig. 13). Importantly, *FHL1* KO mice showed decreased survival in response to IR (Fig. 4e). Kaplan–Meier survival analysis showed that, for breast cancer patients who received radiotherapy, those with tumours that highly expressed FHL1 revealed significantly poorer DFS and OS than those with tumours with low FHL1 expression (DFS: *P*=2.814 × 10^−5^; OS: *P*=2.405 × 10^−4^) (Fig. 4f). In contrast, breast cancer patients who were not treated with radiotherapy had no significant differences in their DFS and OS regardless of the FHL1 amounts in the tumours. Similar trend for DFS was observed in cervical cancer patients (Fig. 4g). The data about OS of the patients with cervical cancer were not available. A multivariate analysis revealed that tumour size, nodal status and FHL1 were independent poor prognostic factors of DFS and OS in breast cancer patients treated with radiotherapy (Supplementary Tables 1 and 2), and tumour size and FIGO stage were independent poor prognostic factors in cervical cancer patients treated with radiotherapy (Supplementary Tables 3 and 4). In contrast, for breast cancer patients who were not treated with radiotherapy, FHL1 failed to be an independent poor prognostic factor of DFS and OS, although nodal status, grade, oestrogen receptor (ER) and HER2 were independent prognostic factors. In cervical cancer patients who were not treated with radiotherapy, no prognostic factors could be identified, possibly due to limited number of samples (Supplementary Tables 3 and 4). These results strongly demonstrate that FHL1 contributes to radiotherapy resistance in cancer.

### Identification of eLIM that interacts with G2/M proteins

**Figure 5:**
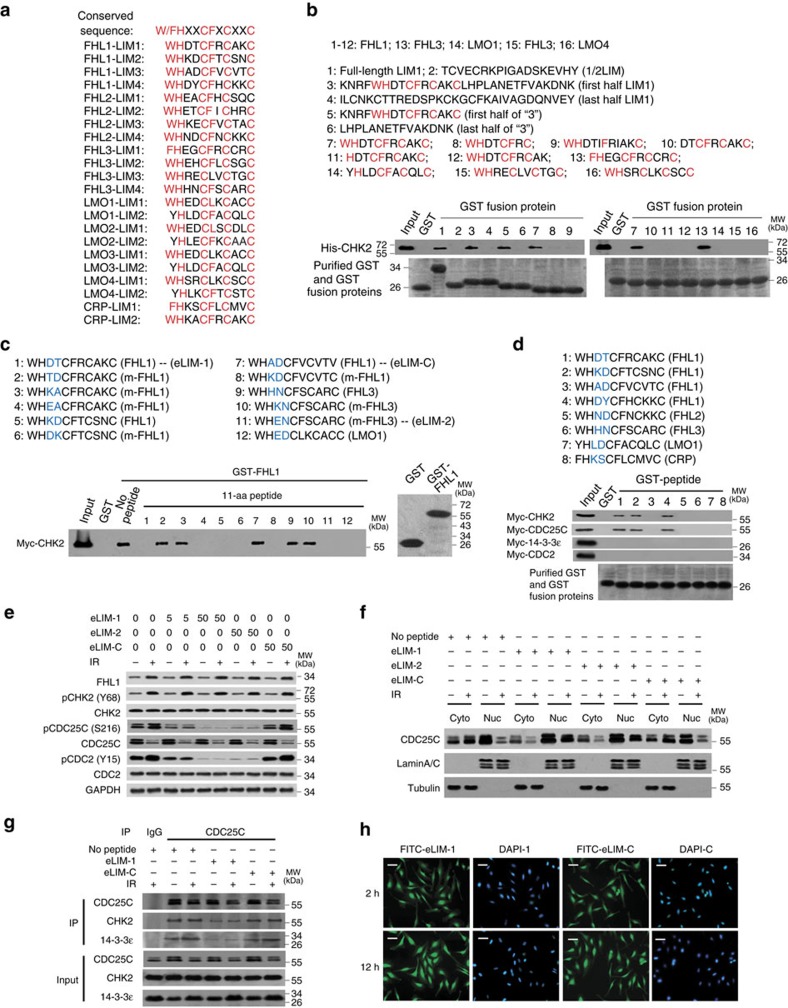
eLIM binds CHK2/CDC25C and promotes phosphorylation and nuclear accumulation of CDC25C. (**a**) Alignment analysis of 11-aa motif in the indicated LIM proteins. Conserved amino acids are marked in red. (**b**) Glutathione-sepharose beads bound with GST or the indicated GST fusion proteins were incubated with purified His-tagged CHK2. After washing the beads, the bound proteins were subjected to SDS–PAGE and immunoblot with anti-His antibody. (**c**) Glutathione-sepharose beads bound with GST-FHL1 were incubated with HeLa cell lysates expressing Myc-tagged CHK2 plus the indicated 11-aa peptides and analysed as in **b**. The two amino acids after the conserved amino acids WH are marked in blue. m-FHLs represent mutant FHLs. (**d**) Glutathione-sepharose beads bound with the indicated GST fusion proteins were incubated with HeLa cell lysates expressing Myc-tagged CHK2, CDC25C, 14-3-3ɛ or CDC2 and analysed as in **b**. (**e**) The eLIM-1, eLIM-2 and eLIM-C peptides (**c**) dissolved in deionized water were added to culture media for HeLa cells at final concentrations of 5 and 50 nM, respectively. Cells were irradiated (8 Gy) and harvested after 6 h of treatment and analysed by immunoblot with the indicated antibodies. The aa sequences of the peptides are shown in **c**. (**f**) HeLa cells were treated with the indicated peptides (50 nM) as in **e**, irradiated (8 Gy), fractionated into cytoplasmic and nuclear fractions, and analysed by immunoblot with the indicated antibodies. (**g**) HeLa cells were treated with the indicated peptides (50 nM) as in **e**, irradiated (8 Gy) and IP with anti-CDC25C or normal IgG, followed by immunoblot with the indicated antibodies. (**h**) HeLa cells were treated with the indicated peptides labelled with FITC (50 nM) at the indicated times. The nuclei were stained with DAPI (blue). The location of the peptides (green) was visualized by a fluorescent microscope. Scale bars, 100 μm.

Since many LIM proteins physically and functionally interact with multiple G2/M-related proteins, we hypothesized that a conserved motif might exist in LIM proteins. Through alignment analysis of aa sequences of LIM domains (http://web.expasy.org/sim), we found that a potential 11-aa motif, namely W/FHχχCFχCχχC (χ may represent any aa), repeatedly appeared in each LIM domain (Fig. 5a). We then determined whether this potential motif interacted with CHK2. Some but not all purified GST fusion proteins containing the potential 11-aa motif (the peptides 1, 3, 5, 7 and 13 but not 14–16) bound purified CHK2 (Fig. 5b), and GST alone and the GST fusion proteins that lack the potential 11-aa motif (the peptides 2, 4 and 6) or contains partial potential 11-aa motif (the peptides 8–12) did not bound CHK2 (Fig. 5b), suggesting that the 11-aa motif is essential for its interaction with CHK2. We noticed that one of the two aa residues after the residues W/FH is often an acidic amino acid (D or E). Competition binding assays using synthesized wild-type or mutated 11-aa peptides indicated that the peptides in which the first aa after W/FH is D or E or the first aa after W/FH is a basic aa followed by an acidic amino acid could compete with full-length FHL1 for binding CHK2, while the other peptides in which the first aa is not an acidic aa or the first aa after W/FH is a basic aa that was not followed by an acidic amino acid could not (Fig. 5c). Thus, the deduced binding motif (eLIM) is W/FHχψCFϕCϕϕC (χ represents an acidic aa or a basic acid followed by an acidic aa, ψ represents any aa or an acidic aa and ϕ is any aa). Except LMO4 and CRP, FHL1-3 and LMO1-3 fit with this motif.

Next, we determined whether eLIM interacts with other G2/M-related proteins. The sequences that are in accordance to eLIM interacted with CDC25C and CHK2, but not 14-3-3ɛ and CDC2 (Fig. 5d). The reason why the sequences that are in accordance to eLIM did not bind 14-3-3ɛ might be due to lack of posttranslational modification of eLIM as mentioned in Fig. 1g or requirement of other aa sequences. Importantly, eLIM-1 and eLIM-2 derived from FHL1 and FHL3, respectively, which are in accordance to eLIM, but not eLIM-C derived from FHL1, which is not in accordance to eLIM, decreased phosphorylation of CDC25C (S216) and CDC2 (Y15) in HeLa and MCF-7 cells (Fig. 5e and Supplementary Fig. 14a). Moreover, eLIM-1 and eLIM-2, but not eLIM-C, increased nuclear accumulation of CDC25C (Fig. 5f and Supplementary Fig. 14b). Mechanistically, eLIM-1 reduced the CHK2–CDC25C and CDC25C–14-3-3ɛ interaction (Fig. 5g). eLIM-1 and eLIM-2, but not eLIM-C, blocked the interaction of FHL1 with CHK2, CDC25C and 14-3-3ɛ (Supplementary Fig. 14c–e). Consistent with this, eLIM-1, eLIM-2 or eLIM-C alone was localized in the nucleus and cytoplasm of HeLa and MCF-7 cells (Fig. 5h and Supplementary Fig. 14f), indicating that the 11-aa motif is cell-penetrating.

### eLIM increases tumour radiosensitivity via the G2 checkpoint

**Figure 6:**
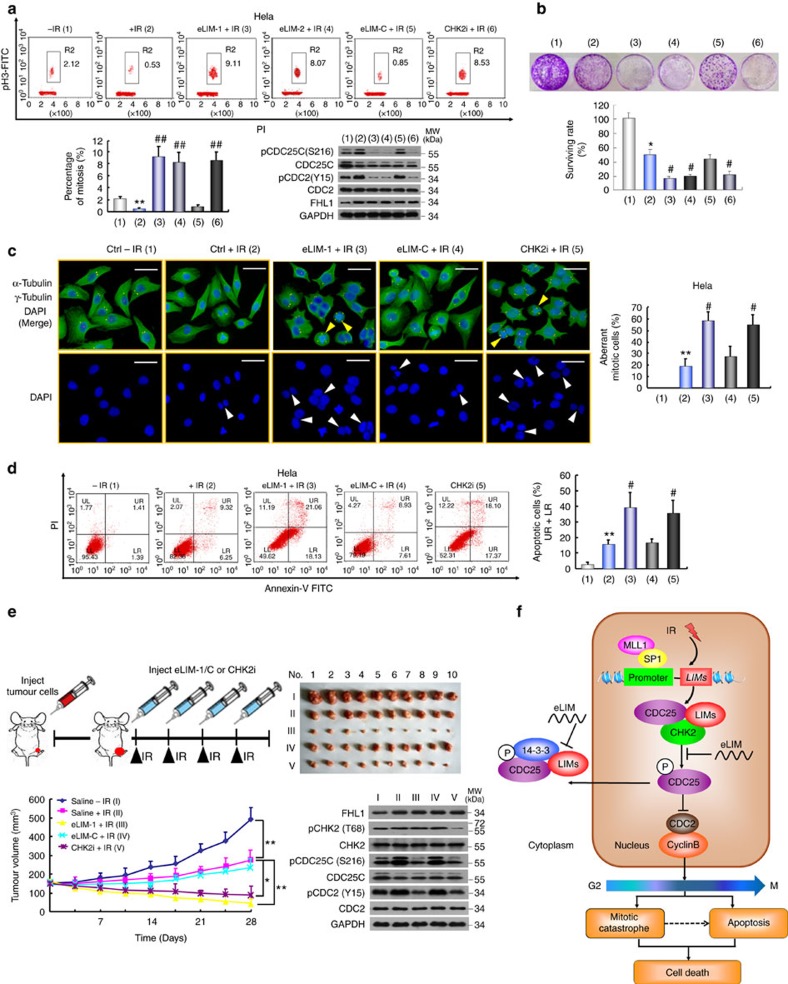
eLIM increases cancer cell radiosensitivity via mitotic catastrophe and apoptosis. (**a**) HeLa cells were treated with the indicated peptides (50 nM), irradiated (8 Gy) and analysed for the proportion of mitotic cells by FACS as in Fig. 4a. Representative FACS and immunoblot with the indicated antibodies are shown. CHK2i, CHK2 inhibitor II (100 nM). (**b**) Clonogenic survival assays of HeLa cells treated as in **a**. (**c**) Immunofluorescence analysis of HeLa cells treated with the indicated peptides or CHK2i and irradiated. Cells were stained with anti-α-tubulin (green) and anti-γ-tubulin (red). The nuclei were stained with DAPI (blue). If green and red colours overlap, yellow colour appears, and if red colour overlaps with blue colour, purple appears. Yellow arrows indicate cells with multipolar spindles and white arrows indicate cells with polymorphologic nucleus. The percentages of aberrant mitotic cells were plotted. Scale bars, 100 μm. (**d**) Representative FACS analysis of apoptosis stained with Annexin V and PI in HeLa cells treated as in **c**. Data shown are mean±s.d. of three independent experiments. *P<0.05, **P<0.01 versus no peptide without IR, ^#^*P*<0.05, ^##^*P*<0.01 versus no peptide with IR (**a**–**d**). (**e**) Volume of xenograft tumours derived from HeLa cells treated with the indicated peptide or CHK2i as indicated. The different number (1–10) refers to different mice. Data are shown as mean±s.d. (*n*=10) (*P<0.05, **P<0.01 at 28 days). Representative tumour tissues (No. 5) were subjected to immunoblot analysis with the indicated antibodies. The *P* values were generated using two-tailed Student's *t*-test (**a**–**e**). (**f**) Proposed model for LIM proteins-mediated modulation of the G2/M checkpoint and radioresistance. IR induces the expression of LIM proteins (For instance, IR stimulates FHL1 transcription in a SP1- and MLL1-dependent manner). LIM proteins increase inhibitory CDC25 phosphorylation by forming a complex with CHK2 and CDC25, and sequesters CDC25 in the cytoplasm by forming another complex with 14-3-3 and CDC25, leading to inactivation of the CDC2/cyclin B complex and subsequent G2/M arrest. eLIM blocks these processes, resulting in mitotic catastrophe and apoptosis.

As eLIM plays an important role in regulation of CDC25 activity, we examined the effects of eLIM on regulation of the G2 checkpoint. Like CHK2 inhibitor, eLIM-1 and eLIM-2 abrogated IR-induced G2/M arrest in HeLa and MCF-7 cells (Fig. 6a and Supplementary Fig. 15a). eLIM-C had little effect. It is well established that cells that have a defective G2 checkpoint enter mitosis before repairing their DNA, leading to cell death by mitotic catastrophe, an event in which a cell with abnormal nucleus is destroyed during mitosis^35^,^36^. The observation that eLIM abrogates the G2 checkpoint suggests that eLIM may result in decreased cell survival in response to IR. Indeed, colony formation assay indicated that eLIM-1, eLIM-2 and CHK2 inhibitor, but not eLIM-C, had radiosensitizing effects in HeLa and MCF-7 cells (Fig. 6b and Supplementary Fig. 15b), accompanied by mitotic catastrophe and apoptosis (Fig. 6c,d and Supplementary Fig. 15c,d). eLIM-1 had less effect in the normal mammary cell line MCF-10A than that in MCF-7 cells (Supplementary Fig. 15e). The radiosensitizing effects of eLIM-1 were also observed in nude mice bearing HeLa or MCF-7 tumours after treatment with intratumoral injection of eLIM-1 in combination with IR (Fig. 6e and Supplementary Fig. 16). The tumours treated with eLIM-1 had reduced phosphorylation of CDC25C (S216) and CDC2 (Y15). Although the volume of tumours treated with IR and eLIM-1 or CHK2i is much smaller than that with other treatments, the levels of pCDC25C (S216) and pCDC2 (Y15) are only moderately altered. This may be because the tumour tissues used for immunoblot were obtained after treatment for a period of time and/or the tumours with dramatically altered levels of pCDC25C (S216) and pCDC2 (Y15) might have been eradicated.

## Discussion

Radiotherapy is an effective treatment that is used to cure or control symptoms of many cancers, such as breast, cervical, prostate, oesophageal and lung cancer^1^,^2^,^3^,^4^,^5^. However, tumour radioresistance is a significant clinical problem. Thus, the elucidation of molecular mechanisms underlying radioresistance would be of great clinical benefit. Cell cycle checkpoint proteins are responsible for the radioresistance of cancer cells. In this study, we have identified a novel class of IR-inducible LIM proteins that regulate the G2/M checkpoint through fine-tune regulation of CDC25 activity (Fig. 6f). CDC25C plays a key role in LIM protein modulation of radioresistance. Recently, CDC25C has been shown to be an independent predictor of better OS for oesophageal squamous cell carcinoma patients treated with radiotherapy^37^. Conceivably, like knockdown of LIM proteins, CDC25C overactivation in oesophageal cancer cells might abrogate the G2 checkpoint in response to IR, leading to decreased cell survival. Very recently, FHL2 knockdown has been shown to increase the radiosensitivity of pancreatic cancer cells *in vitro*^38^. FHL2 knockdown enhances the expression of cyclin B, the component of the M-phase promoting factor CDC2/cyclin B, but whether cyclin B plays a role in FHL2 modulation of radiosensitivity is unknown. We found that knockdown of LIM proteins, such as FHL1-3, also promoted cyclin B expression (data not shown). The mechanism underlying cyclin B expression regulated by LIM proteins and the role of cyclin B in regulation of LIM proteins-mediated radioresistance remain to be investigated.

The LIM superclass of genes can be classified into 15 classes: ABLIM, CRP, ENIGMA, EPLIN, FHL, LASP, LHX, LMO, LIMK, LMO7, MICAL, PXN, PINCH, TES and ZYX^10^,^39^,^40^. Some LIM genes are expressed in a cell- and tissue-specific manner. For example, FHL4 and FHL5/ACT, members of the FHL family, are specifically expressed in the testis. Knockdown of any single FHL protein in cancer cells does not affect the levels of the other FHL proteins, suggesting that no functional redundancy or compensation among the closely related FHL proteins exists^15^. A typical LIM domain contains ∼55 amino acids with 8 highly conserved (cysteine/histidine) residues at defined intervals. The LIM domain mediates protein–protein interactions. We have narrowed down the LIM domain and identified an 11 amino-acid motif within the LIM domain called eLIM. The eLIM is sufficient for interaction with CHK2 and CDC25. We also found that the eLIM interacted with other G2/M-related proteins (data not shown). These data suggest that the eLIM is critical for mediation of protein–protein interactions. In addition, we cannot exclude the possibility that the eLIM can associate with other proteins besides G2-M proteins. Like the BRCT and FHA domains^41^,^42^, the eLIM domain may also play a critical role in regulation of DNA damage response. Intriguingly, the 11-aa peptides are cell-penetrating. The previous observation that purified recombinant FHL3 protein added to cell culture media regulates cancer cell growth might be explained by the fact that FHL3 contains four 11-aa motifs. Thus, the 11-aa peptides may be used to deliver exogenous molecules into cells.

Most cancer cells show impaired modulation at their cell cycle G1 checkpoint. This is due to abnormalities in classic oncogenes and in tumour suppressors, such as ras, c-myc, p53 and Rb^43^,^44^. The unique dependency of most cancer cells on G2 checkpoint to survive with DNA damage makes G2 checkpoint abrogation an attractive strategy for sensitizing cancer cells to DNA-damaging anticancer agent without increasing adverse effects on normal cells. CHK1 and CHK2 inhibitors are being tested in phase II clinical trials for their ability to abrogate G2 checkpoint function and to sensitize cancer cells to DNA-damaging agents^45^. These inhibitors in combination with DNA-damaging agents cause loss of phospho-CDC25C (S216) and accumulation of CDC25C in the nucleus via inhibition of 14-3-3 binding to CDC25C, thereby abrogating the G2 checkpoint and inducing mitotic catastrophe. Like CHK2 inhibitor, the cell-penetrating eLIM peptide abrogates the G2 checkpoint through reduced phospho-CDC25C (S216) and increased nuclear CDC25C after exposure of cancer cells to IR, causing mitotic catastrophe and apoptosis. Thus, the eLIM peptides may be novel anticancer drug candidates with G2 checkpoint-abrogating activity.

## Methods

### Plasmids and lentiviruses

The eukaryotic expression vectors for FLAG-tagged FHL1, FHL2, FHL3 and siRNA-resistant FHL1 have been described previously^15^. The FHL1 promoter luciferase reporters were made by inserting PCR-amplified promoter fragments from genomic DNA into the pGL4-Basic vector (Promega). Other mammalian expression vectors encoding FLAG-, MYC- or HA-fusion proteins tagged at the amino terminus were constructed by inserting PCR-amplified fragments into pcDNA3 (Invitrogen). Plasmids encoding GST fusion proteins were generated by cloning PCR-amplified sequences into pGEX-KG (Amersham Pharmacia Biotech). Lentiviral vectors for gene overexpression were constructed by inserting PCR-amplified gene fragments into pCDH (System Biosciences). Lentiviral shRNA vectors were made by cloning short hairpin RNA fragments into pSIH-H1-Puro (System Biosciences). The number of the NCBI Reference Sequence for genes used in this study is shown in Supplementary Table 5. siRNAs for CDC25A, CDC25B, CDC25C, SP1, MLL1 (GenePharma Company, Shanghai) and 14-3-3ɛ (Invitrogen, Carlsbad, CA, USA) with reported sequences were chemically synthesized (Supplementary Table 6). Lentiviruses were produced by cotransfection of HEK293T cells with recombinant lentivirus vectors and pPACK Packaging Plasmid Mix (System Biosciences) using Megatran reagent (Origene). Lentiviruses were collected 48 h after transfection and added to the medium of target cells with 8 μg ml^−1^ polybrene (Sigma-Aldrich). Stable cell lines were selected in 1 μg ml^−1^ puromycin for ∼2 months. Pooled clones or individual clones were screened by standard immunoblot protocols and produced similar results.

### Peptides and reagents

Peptides or peptides conjugated with FITC (>95% purity) were synthesized by SBS Genetech Company (Beijing).

Anti-Myc (sc-40HRP, 1:5,000), anti-phos-ERK1/2 (Y204) (sc-7383, 1:500), anti-SP1 (sc-16646-R, 1:1,000), anti-CDC25B (sc-5619, 1:200), anti-CDC25C (sc-13138, 1:500), anti-phospho-CDC25C (S216) (sc-12354-R, 1:200), anti-CDC2 (sc-137035, 1:1,000), anti-phos-CDC2 (Y15) (sc-7989-R, 1:500), anti-CHK2 (sc-17747, 1:500), anti-CHK2 (sc-9064, 1:250), anti-phospho-CHK2 (T68) (sc-16297-R, 1:100), anti-14-3-3ɛ (sc-292984, 1:500), anti-14-3-3ɛ (sc-23957, 1:200) and anti-Lamin A/C (sc-20681, 1:1,000) antibodies were purchased from Santa Cruz Biotechnology; anti-FLAG (A8592, 1:5,000), anti-FLAG M2 agarose (A2220), anti-GFP (G1546, 1:2,000) and anti-GAPDH (G9295, 1:10,000) were obtained from Sigma-Aldrich; anti-phospho-CDC25A (S178) (ab79252, 1:200), anti-phospho-CDC25B (S323) (ab53103, 1:200), anti-H3K4me (ab8895, 1:1,000) and anti-SETD7 (ab14820, 1:1,000) were purchased from Abcam; anti-H3K4me2 (17-677, 1:1,000) and anti-H3K4me3 (17–678, 1:1,000), anti-CHK2 (05-649, 1:500), anti-CDC25A (05-743, 1:500) and anti-phospho-Histone H3 (Ser10) (06-570, 1:100) were from Millipore; anti-SET1A (A300-289A, 1:500) and anti-MLL1 (A300-374A, 1:200) were purchased from Bethyl; anti-CDC25C (16485-1-AP, 1:500), anti-FHL1 (10991-1-AP, 1:500), anti-FHL2 (21619-1-AP, 1:1,000), anti-FHL3 (11028-1-AP, 1:500), α-Tubulin (11224-1-AP, 1:1,000) was from Proteintech; anti-GST (RPN1236, 1:10,000) and anti-His (27471001, 1:10,000) antibodies were purchased from GE Healthcare Life Sciences.

### *FHL1* KO mice

*FHL1* KO C57BL/6J mice were generated by TALEN-mediated gene targeting (Shanghai Model Organisms Center). The target sequences of the *FHL1* TALENs were as follows: 5′-TCGACTGTCACTACT-3′ (left) and 5′-GCACGTACTTCTTCCCCT-3′ (right). The TALEN mRNAs were microinjected into oocytes. Genomic DNA was prepared from the tail tips of newborn mice and the *FHL1* mutation was identified by PCR amplification of genomic DNA, DNA sequencing and immunoblot.

### KO cells

CRISPRs (clustered regularly interspaced short palindromic repeats) were designed using a CRISPR design web tool (http://crispr.mit.edu). The sgRNA (single guide RNA) sequences efficiently targeted by *FHL1*, *CDC25C* and *14-3-3ɛ* CRISPRs are CTACTGCAGGGATCCCTTGC, CGATGCCAGAGAACTTGAAC and GTTGCATATAAGAATGTGAT, respectively. The sgRNAs were cloned into the pGK1.1/CRISPR/Cas9 vector (Genloci Biotechnologies) according to the manufacturer's instructions. Cells were transfected with the sgRNA vectors, expanded and screened for mutations at nuclease target sites by PCR amplification of genomic sequences, followed by DNA sequencing and immunoblotting. MEFs were isolated from *FHL1* KO and *CDC25C* KO (Jackson Laboratory; Stock No. 009364) mouse embryos at day 14 of gestation^46^.

### Cell culture and transfection

Human cervical cancer HeLa cells, human breast cancer MCF-7, ZR75-1, MD-MBA-231 cells and normal human mammary MCF-10A cells were purchased from American Type Culture Collection (ATCC), and have previously been tested for mycoplasma contamination. Cells were routinely cultured in DMEM (Invitrogen) containing 10% FBS (Hyclone). The IR-resistant Eca109 cells and its parental cells were obtained from Dr Rui Liu (Xi'an Jiao Tong University, China)^47^. Lipofectamine 2000 reagent and Lipofectamine RNAiMAX were used for transfections of plasmids and siRNAs, respectively, according to the manufacturer's guidelines (Invitrogen).

### Luciferase assay

Cells were seeded in 24-well plates and transfected with 0.2 μg of promoter reporter, 50 ng of expression vector and 0.1 μg of β-galactosidase reporter. After exposed to 10 Gy irradiation, the transfected cells were harvested and luciferase and β-galactosidase activities were determined. β-Galactosidase activity was used as an internal control for transfection efficiency^48^.

### Yeast two-hybrid

The bait plasmid pAS2-FHL1 and a human mammary two-hybrid cDNA library (Clontech) were sequentially transformed into *Saccharomyces cerevisiae* strain CG1945 according to the manufacturer's instructions (Clontech). Transformants were grown on synthetic medium lacking tryptophan, leucine and histidine but containing 1 mM 3-aminotriazole. The candidate clones were rescued from the yeast cells and re-transformed back to the same yeast strain to confirm the interaction between the candidates and the bait. The specificity of the interaction was determined by comparing the interactions between the candidates and various bait constructs. The unrelated bait plasmid pAS2-lamin C was used as a negative control.

### GST pull-down and co-immunoprecipitation assays

For GST pull-down assay, GST or His fusion proteins were expressed and purified according to the manufacturers' instructions (Amersham Pharmacia and Qiagen). Purified His fusion proteins or cell lysates were incubated with GST fusion protein bound to GST beads for 4 h at 4 °C. After washing, the precipitated components were analysed by immunoblot. For co-immunoprecipitation (Co-IP) assay^49^, cells were lysed in 0.5 ml lysis buffer (50 mM Tris at pH 8, 500 mM NaCl, 0.5% Nonidet P-40, 1 mM dithiothreitol and protease inhibitor tablets from Roche Applied Science) and immunoprecipitated (IP) with antibody or control serum (Santa Cruz). After extensive washing with the lysis buffer, the immunoprecipitates were resolved by SDS–PAGE, followed by western blot analysis.

### Fast protein liquid chromatography

Cells were prepared and dialyzed against buffer D (20 mM HEPES, pH 8, 10% glycerol, 0.1 mM EDTA, 300 mM NaCl). Approximately 1 ml volume of cell lysate was separated by an ÄKTA pure chromatography system (Amershan Biosciences) using a Superose 6 10/300GL column (GE Healthcare Life Sciences) that had been equilibrated with buffer D and calibrated with protein standards (Gel Filtration Calibration Kits, GE Healthcare Life Sciences, blue dextran, 2,000 kDa; thyroglobulin, 669 kDa; ferritin, 440 kDa; catalase, 158 kDa; bovine serum albumin, 75 kDa). The column was eluted at a flow rate of 0.5 ml min^−1^ and fractions were collected.

### Subcellular fractionation

The localization of proteins was determined by subcellular fractionation^49^. Briefly, cells were homogenized using a Dounce homogenizer, and the homogenate was centrifuged at 366*g* for 10 min. The pellet was analysed as the nuclear fraction. The supernatant was centrifuged again at −16,200 *g* for 10 min, and the final supernatant was analysed as the cytoplasmic fraction.

### Real-time reverse transcription-PCR

Total RNA was extracted using TRIzol reagent according to the manufacturer's protocol (Invitrogen). RNA was reverse transcribed into cDNA by Quantscript RT Kit (Tiangen). Real-time PCR was performed with primers listed in Supplementary Table 7.

### Chromatin immunoprecipitation and re-ChIP

ChIP assay was performed using the Magna ChIP Assay Kit (Millipore) according to the manufacturer's instructions. For re-ChIP, complexes were eluted from the primary immunoprecipitation by incubation with 10 mM DTT at 37 °C for 30 min and diluted 1:50 in re-ChIP buffer (150 mM NaCl, 1% Triton X-100, 2 mM EDTA, 20 mM Tris–HCl, pH 8.1) followed by re-immunoprecipitation with the second antibodies. Real-time PCR was performed to detect relative occupancy. The primers used for real-time PCR are displayed as follows: *FHL1* promoter forward: 5′-GATGGGGCTTATTTAGCTCCCTC-3′; *FHL1* promoter reverse: 5′-CTTCGGGGCCCACGCCGTTT-3′; *FHL1* upstream forward: 5′-AAGGACTTGATTACTTTGTGTGCTG-3′; *FHL1* upstream reverse: 5′-AGCACGAGGAAAACGGCCTTC-3′.

### Kinase assays

Immune complexes with anti-CDC2 were incubated with purified GST-Histone H1 in kinase buffer (50 mM HEPES, 10 mM MgCl_2_, 1 mM DTT, 2.5 mM EGTA, 0.1 mM Na_3_VO_3_, 1 mM NaF) containing γ-^32^P-ATP for 30 min at 37 °C. The reaction products were analysed by SDS–PAGE and autoradiography.

### Colony formation assay

Cells were irradiated with the indicated doses using a cobalt-60 γ-ray source at a dose rate of 1.7 Gy min^−1^ and cultured for 10–14 days. Cells were stained with crystal violet and colonies consisting of 50 or more cells were counted. The survival fraction was calculated as the mean number of colonies/(cells seeded × plating efficiency).

### Flow cytometry analysis

Cell apoptosis was detected using an Annexin V/PI staining kit (BD Biosciences) according to the manufacturer's protocol. Mitotic cells were detected using anti-phospho-histone H3 (Ser10) (1: 50) and PI double staining.

### Mouse irradiation study

Animal studies were performed in accordance with protocols approved by the Institutional Animal Care and Use Committee at Beijing Institute of Biotechnology. Five million tumour cells were injected into the abdominal mammary fat pad (for breast cancer) or the muscle of the right hind limb (for cervical cancer) of 6-week-old female BALB/c nude mice. When tumours reached the volume of approximately 150 mm^3^, we randomly allocated the mice to groups in which they received placebo (normal saline), peptides or CHK2i with or without IR as indicated. We determined randomization using SPSS 13.0 statistical software. Before IR treatment, peptides dissolved in normal saline (100 μl per mouse) were intratumorally injected. Four hours after injection with 200 μg kg^−1^ of peptides, the mice were subjected to IR. Injection combined with IR was performed once per week till the end point. Single IR dose (10 cGy once) was delivered to the tumours using a cobalt-60 γ-ray source at a dose rate of 101.8 cGy min^−1^. CHK2i (100 μg kg^−1^) was used like peptides. Tumour growth was determined by caliper measurements. Tumour volume was calculated according to the following formula: volume=(longest diameter × shortest diameter^2^)/2. Excised tumours were weighed, and portions were frozen in liquid nitrogen or fixed in 4% paraformaldehyde for further study. Similar experiments done previously were used to estimate sample size.

For *FHL1* wild-type and KO mice, 4-week-old female C57BL/6J mice were exposed to cobalt-60 γ-ray source for 10 Gy radiation (whole-body single exposure) at a dose rate of 90.85 cGy min^−1^ and examined for survival. The wild-type mice used for the experiment were littermates of the KO mice and sixteen independent KO mouse lines were analysed.

### Clinical samples and IHC

Two hundred and twenty-six cases of primary breast carcinomas (tissue microarray) and seventy nine cases of cervical cancer were obtained from Chinese PLA General Hospital, with the informed consent of patients and with the approval of the Institutional Review Committees of Chinese PLA General Hospital. Similar experiments performed previously were used to estimate sample size. All cases were female with 26–84 years of age (mean age: 51.3 years) for breast cancer and 22–78 years of age (mean age: 49.6 years) for cervical cancer. The follow-up time was 1–120 months (mean: 69.3 months) for breast cancer and 1–79 months (mean: 36 months) for cervical cancer. Normal distribution was performed using SPSS13.0. For IHC, formalin-fixed paraffin-embedded samples were used^50^. The samples were deparaffinized, rehydrated and pretreated with 3% hydrogen peroxide for 20 min to quench endogenous peroxidase activity. The antibody-binding epitopes of the antigens were retrieved by microwave treatment, and the samples were then preincubated with 10% normal serum to block nonspecific binding. Rabbit anti-FHL1 (10991-1-AP, Proteintech), rabbit anti-CDC25C (16485-1-AP, Proteintech) and rabbit anti-phospho-CDC25C (S216) (sc-12354-R, Santa Cruz Biotechnology) were used at dilutions of 1: 100, 1: 100 and 1: 50, respectively, as the primary antibodies for IHC in breast cancer. The same anti-FHL1 was used at a dilution of 1:50 for IHC in cervical cancer. The specimens were incubated with the primary antibodies for 1 h at room temperature, followed by the addition of biotinylated anti-rabbit secondary antibody and streptavidin-horseradish peroxidase. 3,3′-Diaminobenzidine was used as a chromogen and hematoxylin was used for counterstaining. The FHL1, CDC25C or phospho-CDC25C (S216) score was generated by multiplying the percentage of stained cells (0–100%) by the intensity of the staining (low, 1+; medium, 2+; strong, 3+). Thus, the score is between 0–3. The optimal cutoff value of the IHC scores were determined using receiver operating characteristic (ROC) curve analysis^51^. We defined score ≤0.75 as low FHL1, CDC25C or phospho-CDC25C (S216) and score >0.75 as high FHL1, CDC25C or phosphor-CDC25C (S216).

### Statistical analysis

Trial experiments or similar experiments done previously were used to assess sample size with adequate statistical power. Statistical significance in the preclinical experiments was assessed by two-tailed Student's *t*-test. The correlation of FHL1 with phospho-CDC25C (S216) was determined using the Spearman's rank correlation test. The comparison of nuclear or cytoplasmic staining of CDC25C between FHL1 low and high tissues was determined using the Mann–Whitney *U* test. Estimation of DFS and OS was performed using the Kaplan–Meier method, and differences between survival curves were examined with the log-rank test. All statistical tests were two-sided. Statistical calculations were performed using SPSS 13.0. In all assays, *P*<0.05 was considered statistically significant.

### Data availability

All data in this study are available within the article and its Supplementary Files or available from the authors upon request. Uncropped scans of the most important immunoblots are shown in Supplementary Information (Supplementary Fig. 17).

## Additional information

**How to cite this article:** Xu, X. *et al*. A signature motif in LIM proteins mediates binding to checkpoint proteins and increases tumour radiosensitivity. *Nat. Commun.* **8,** 14059 doi: 10.1038/ncomms14059 (2017).

**Publisher's note:** Springer Nature remains neutral with regard to jurisdictional claims in published maps and institutional affiliations.

## Supplementary Material

Supplementary Information Supplementary Figures, Supplementary Tables and Supplementary References
