# Supplementary material for: A signature motif in LIM proteins mediates binding to checkpoint proteins and increases tumour radiosensitivity
Source: Nat Commun. 2017 Jan 17;8:14059. doi: 10.1038/ncomms14059 (PMC5247581; doi:10.1038/ncomms14059)
Supplement: Supplementary Information — Supplementary Figures, Supplementary Tables and Supplementary References [file ncomms14059-s1.pdf]

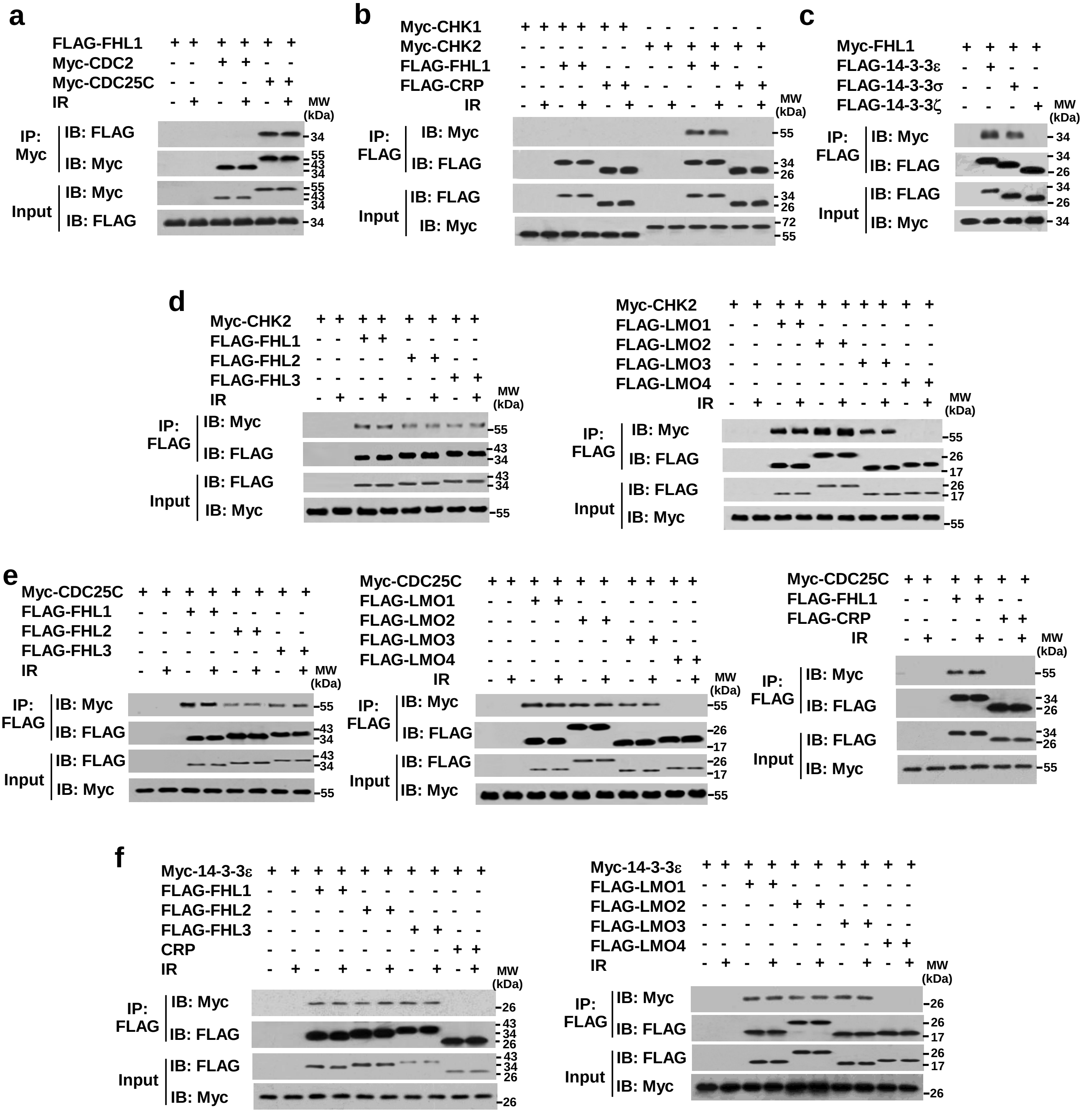

Supplementary Figure 1. LIM proteins interact with CHK2, CDC25C and 14-3-3. (**a**) HeLa cells were transfected with FLAG-tagged FHL1 and Myc-tagged CDC25C or CDC2, and treated with or without IR (8Gy). Six hours later, cell lysates were immunoprecipitated (IP) by anti-Myc antibody, followed by immunoblot (IB) with the indicated antibodies. (**b**) HeLa cells were transfected with FLAG-tagged FHL1 or CRP and Myc-tagged CHK1 or CHK2, and treated with or without IR (8Gy). Six hours later, cell lysates were immunoprecipitated by anti-FLAG antibody, followed by immunoblot with the indicated antibodies. (**c-f**) HeLa cells were transfected with the indicated constructs. Transfected cells were treated and analyzed as in (b). MW, molecular weight (a-f).

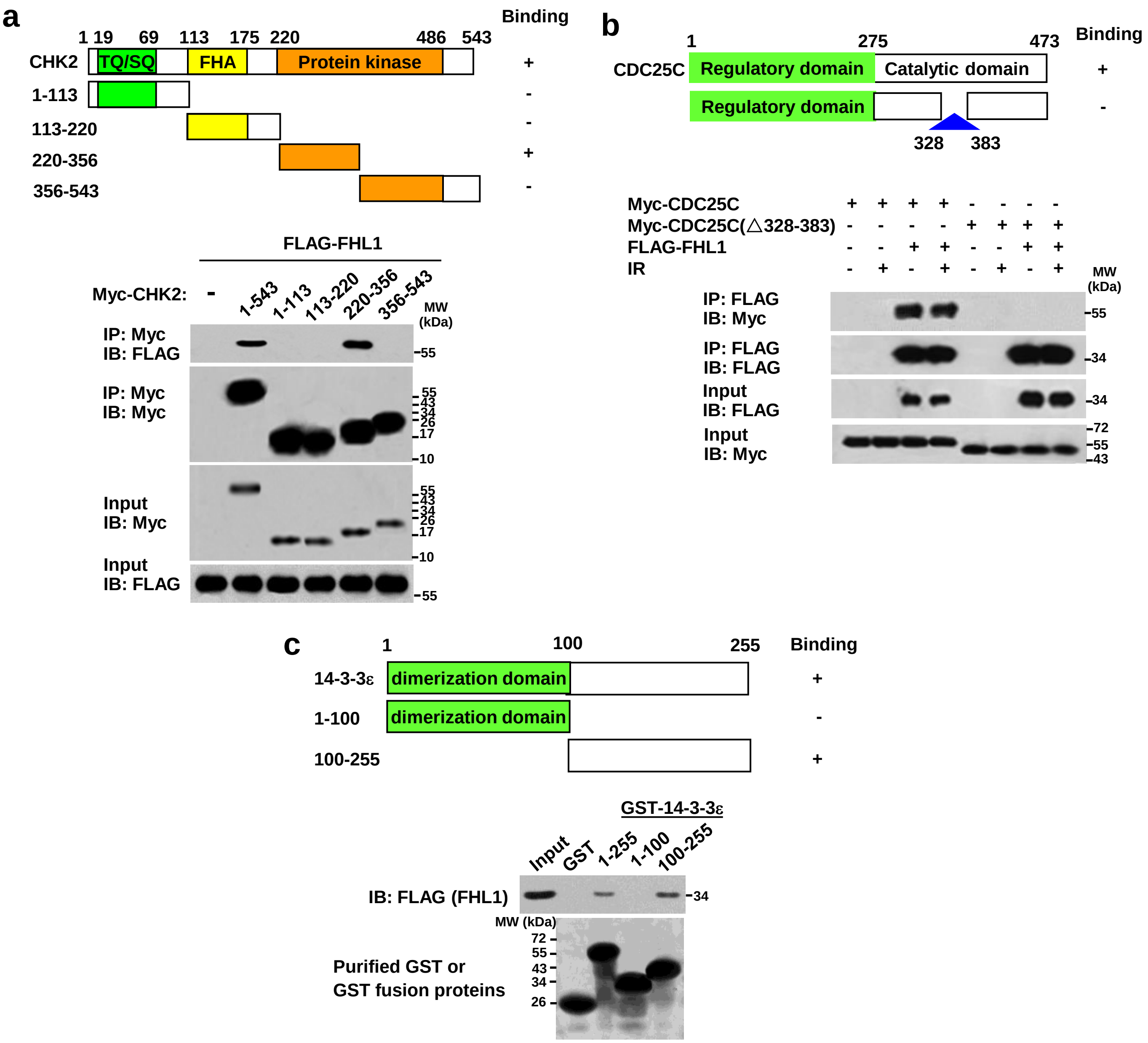

Supplementary Figure 2. Mapping of the region of CHK2, CDC25C and 14-3-3ε that interacts with FHL1. (**a**,**b**) The binding ability of the indicated Myc-tagged CHK2 (a) and CDC25C (b) deletion mutants was analyzed in HeLa cells by Co-IP assays. (**c**) GST pull-down analysis of purified GST or GST-14-3-3ε deletion mutants incubated with lysates of HeLa cells expressing FLAG-tagged FHL1. Schematic diagrams of CHK2, CDC25C, 14-3-3ε and their deletion constructs used are shown at the top (a-c).

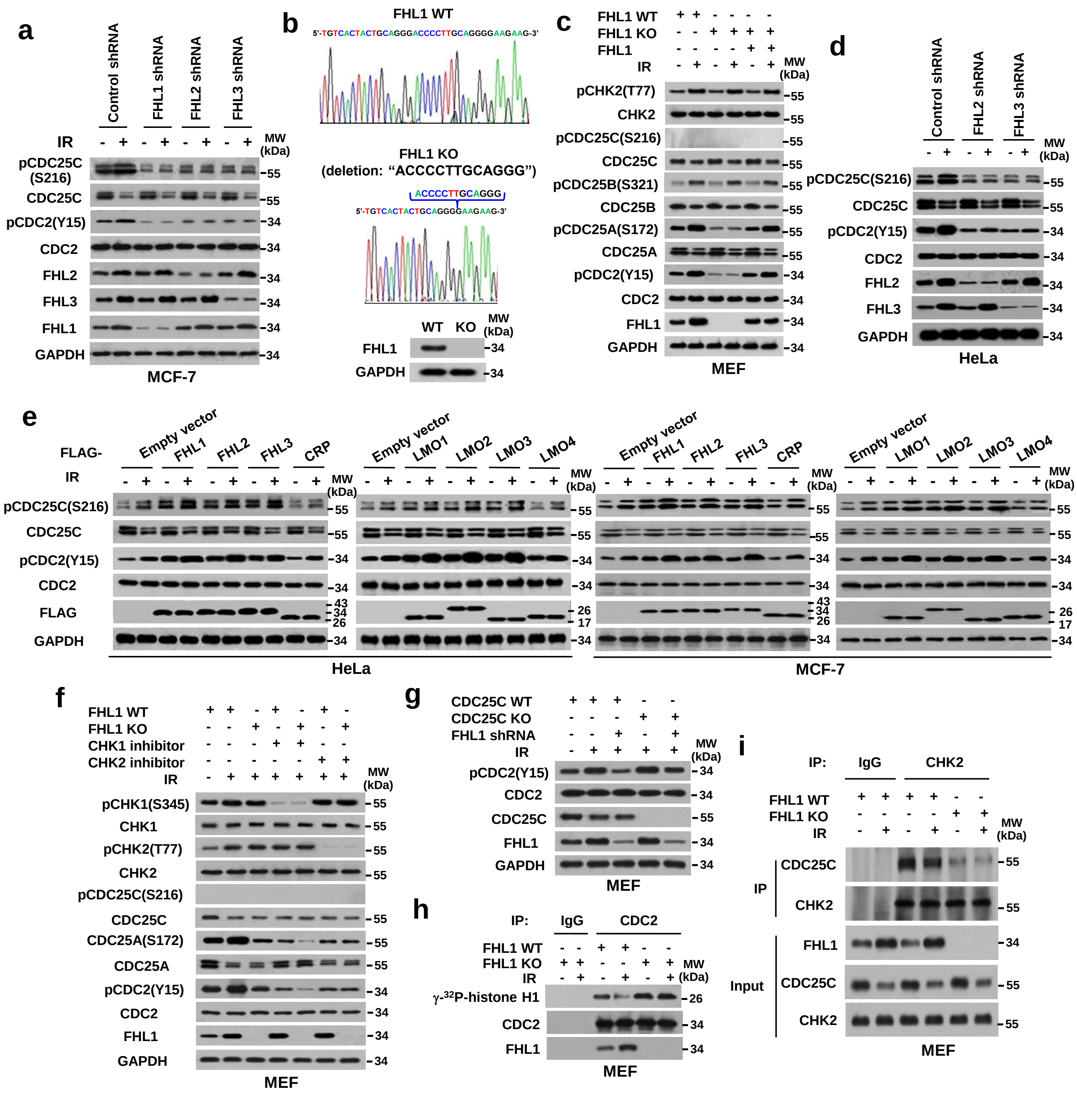

Supplementary Figure 3. **FHL1 regulates CDC25 activity via CHK2-mediated CDC25 phosphorylation. (a)** Immunoblot analysis of MCF-7 cells stably infected with the indicated constructs, exposed to IR (8 Gy) and harvested 6 h after IR. **(b)** Identification of FHL1 knockout mice by DNA sequencingt and immunoblot. **(c)** Immunoblot analysis of FHL1 wild-type (WT) MEFs or FHL1 knockout (KO) MEFs or FHL1 KO MEFs infected with lentivirus carrying FHL1, exposed to IR (8Gy) and harvested 6 h after IR. Mouse pCHK2 (T77), pCDC25B (S321) and pCDC25A (S172) correspond to human pCHK2 (T68), pCDC25B (S323) and pCDC25A (S178) , respectively, and the antibodies against human pCHK2 (T68), pCDC25B (S323) and pCDC25A (S178) could react with mouse pCHK2 (T77), pCDC25B (S321) and pCDC25A (S172), respectively. **(d)** Immunoblot analysis of HeLa cells stably infected with the indicated constructs, exposed to IR (8 Gy) and harvested 6 h after IR. **(e)** Immunoblot analysis of HeLa or MCF-7 cells transiently transfected with FLAG-tagged FHL1-3, LMO1-4 or CRP, exposed to IR (8Gy) and harvested 6 h after IR. **(f)** Immunoblot analysis of FHL1 WT or FHL1 KO MEFs treated with 50 nM CHK1 inhibitor SB-218078 (Tocris) or 100 nM CHK2 inhibitor II (Sigma-Aldrich) and exposed to IR (8 Gy). Cells were harvested 6 h after IR and analyzed with the indicated antibodies. **(g)** CDC25C WT or KO MEFs were infected with lentivirus carrying FHL1 shRNA. Cells were treated with IR (8Gy) and analyzed by immunoblot with the indicated antibodies. **(h)** FHL1 WT or KO MEFs were irradiated and immunoprecipitated with anti-CDC2 or normal IgG. In vitro kinase assays were performed using the immunoprecipitates and the substrate GST-histone H1, and analyzed by SDS-PAGE and immunoblot or autoradiography as indicated. **(i)** FHL1 WT or KO MEFs were irradiated and immunoprecipitated with anti-CHK2 or normal IgG. The precipitates were analyzed by immunoblotting with the indicated antibodies.

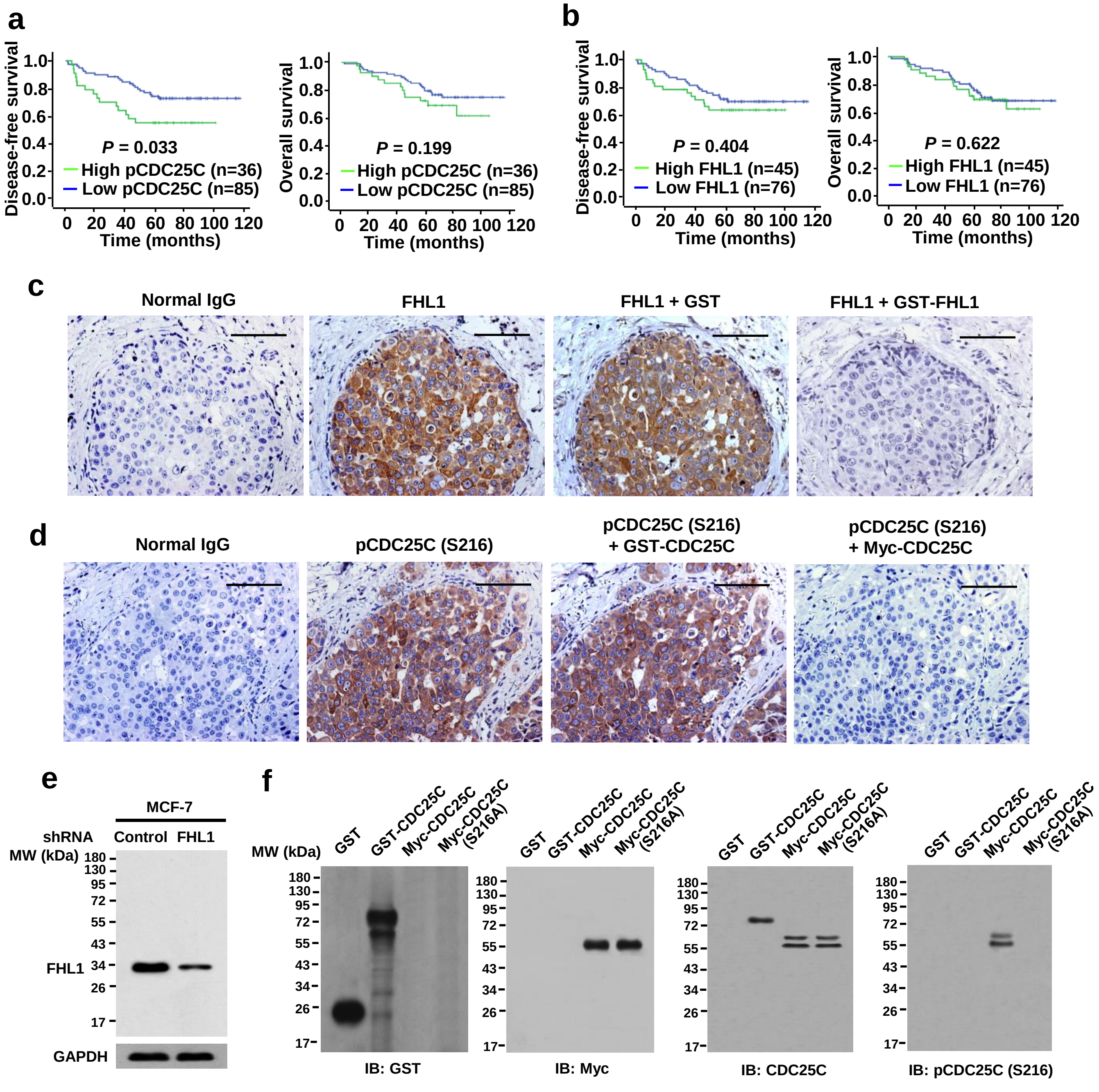

Supplementary Figure 4. Clinical outcome of FHL1 and pCDC25C (S216) expression in breast cancer patients. (a,b) Kaplan-Meier estimates of disease-free survival and overall survival of breast cancer patients with different pCDC25C(S216) (a) and FHL1 expression (b). **(c,d)** Validation of antibody specificity to FHL1 and pCDC25C(S216). Immunohistochemical staining of breast cancer specimens incubated with normal IgG or anti-FHL1. To validate antibody specificity, the anti-FHL1 was pre-incubated with recombinant GST-FHL1 protein or GST for 1 h prior to applying to tissue. Similarly, the anti-pCDC25C (S216) was pre-incubated with purified GST-CDC25C protein (no phosphorylation modification) from Escherichia coli or Myc-tagged CDC25C protein (phosphorylated CDC25C) purified by immunoprecipitation from HEK293T cells transfected with Myc-tagged CDC25C for 1 h prior to applying to tissue. Original magnification, ×20. Scale bar, 100 μm. **(e)** Immunoblot analysis of lysates from MCF-7 cells infected with control shRNA or FHL1 shRNA with anti-FHL1. **(f)** Purified GST and GST-CDC25C from Escherichia coli and Myc-tagged CDC25C or CDC25C (S216A) purified by immunoprecipitation from HEK293T cells transfected with Myc-tagged CDC25C or CDC25C (S216A). Immunoblot was performed with the indicated antibodies.

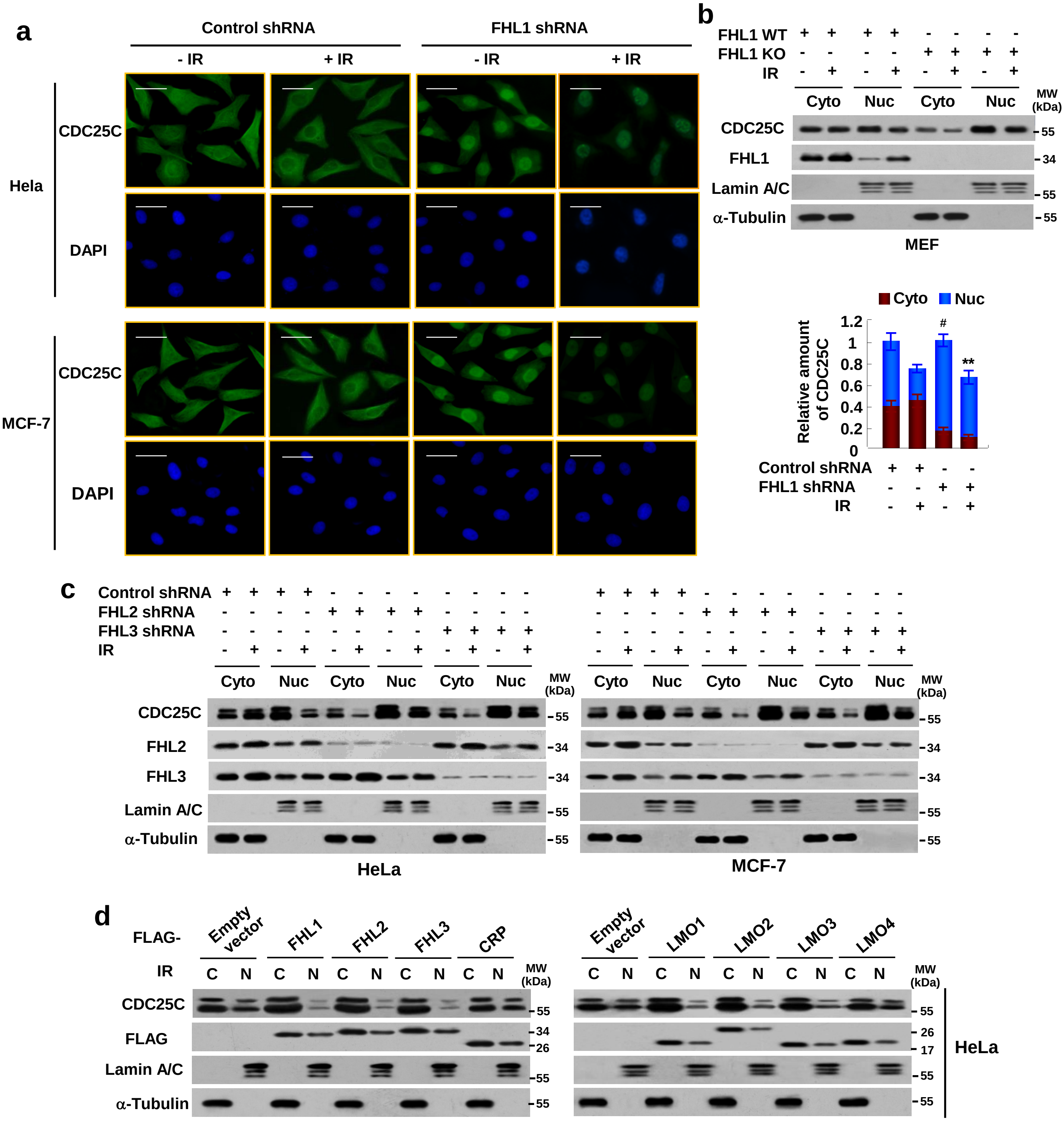

Supplementary Figure 5. LIM proteins sequester CDC25C in the cytoplasm. (**a**) Hela or MCF-7 cells stably infected with FHL1 shRNA and control shRNA were exposed to IR (8Gy). Cells were stained with anti-CDC25C (green). The nuclei were stained with DAPI (blue). Scale bar: 100 μm. **(b)** FHL1 WT or FHL1 KO MEFs were exposed to IR (8Gy), and fractionated into cytoplasmic (Cyto) and nuclear (Nuc) fractions, and analyzed by immunoblotting with the indicated antibodies. Lamin A/C and α-Tubulin were used as the nuclear and cytoplasmic marker, respectively. The densitometric quantitation of CDC25C is shown at the bottom of immunoblots. Values shown are mean ± SD of 3 independent experiments. The *P* value was generated using two-tailed Student's *t*-test. $^{\#}p < 0.05$ versus control shRNA without IR (the ratio of nucleus to cytoplasm), $^{**}p < 0.01$ versus control shRNA with IR. (**c**) HeLa or MCF-7 cells stably infected with the indicated constructs were exposed to IR, fractionated into cytoplasmic and nuclear fractions and analyzed by immunoblot with the indicated antibodies. (**d**) HeLa cells were transiently transfected with FLAG-tagged FHL1-3, LMO1-4 or CRP and exposed to IR. Transfected cells were fractionated and analyzed as in (c).

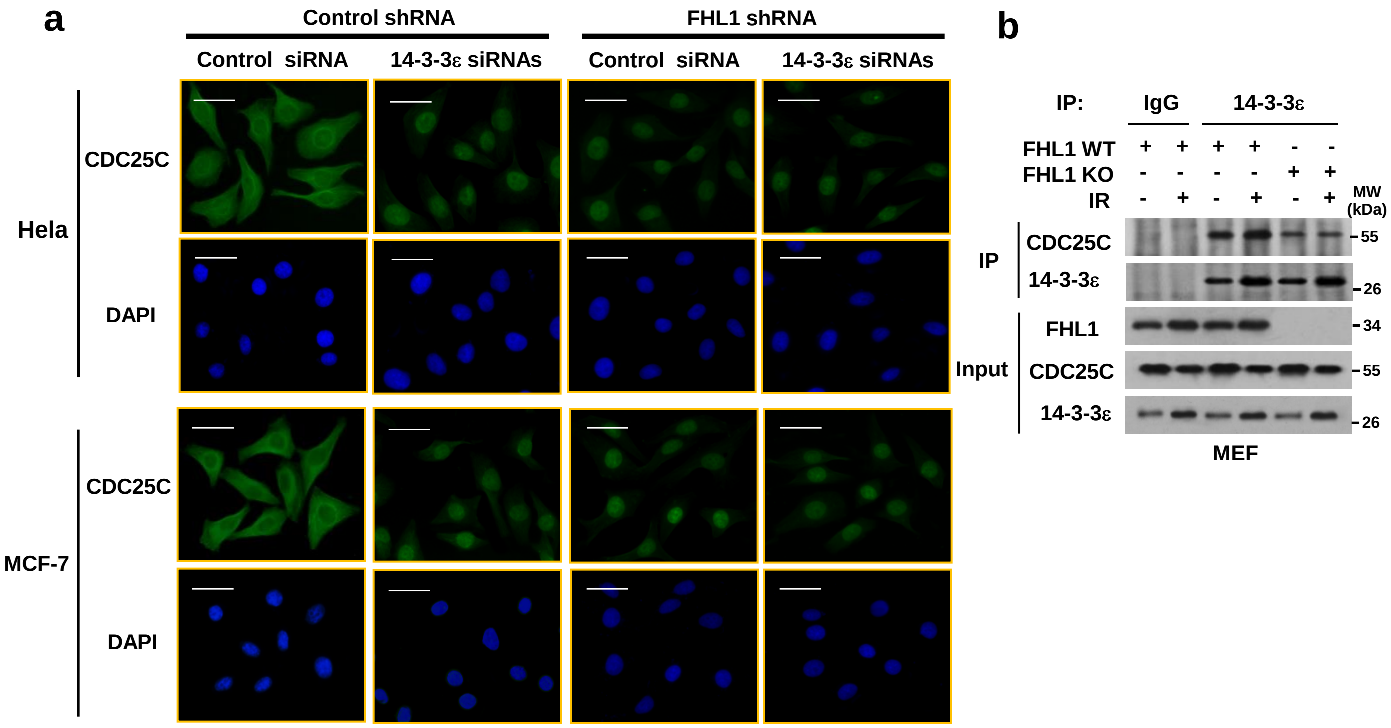

Supplementary Figure 6. FHL1 sequesters CDC25C in the cytoplasm via 14-3-3ε. (**a**) HeLa or MCF-7 cells stably infected with FHL1 shRNA or control shRNA were transfected with 14-3-3ε siRNAs, exposed to IR and analyzed by immuimmunofluorescence. Cells were stained with anti-cdc25C (green). The nuclei were stained with DAPI (blue). Scale bar: 100 μm. **(b)** FHL1 WT or KO MEFs were irradiated and immunoprecipitated with anti-14-3-3ε or normal IgG. The precipitates were analyzed by immunoblotting with the indicated antibodies.

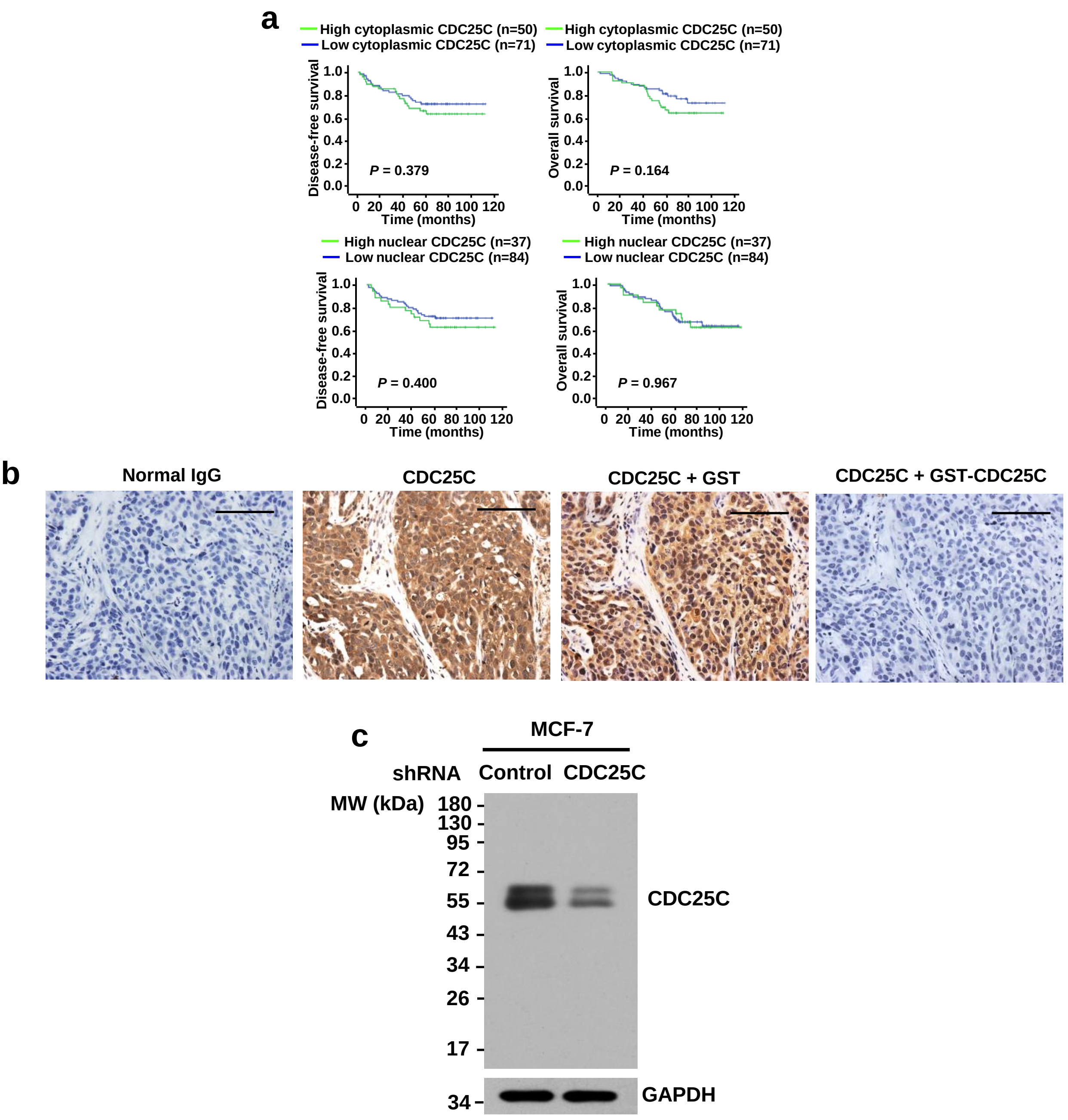

Supplementary Figure 7. Clinical significance of cytoplasmic and nuclear CDC25C expression in breast cancer patients. (a) Kaplan-Meier estimates of disease-free survival and overall survival of breast cancer patients with different cytoplasmic CDC25C and nuclear CDC25C expression. **(b)** Validation of antibody specificity to CDC25C. Immunohistochemical staining of breast cancer specimens incubated with normal IgG or anti-CDC25C. To validate antibody specificity, the anti-CDC25C was pre-incubated with recombinant GST-CDC25C protein or GST for 1 h prior to applying to tissue. Original magnification, ×20. Scale bar, 100 μm. **(c)** Immunoblot analysis of lysates from MCF-7 cells infected with control shRNA or CDC25C shRNA using antibodies specific for anti-CDC25C.

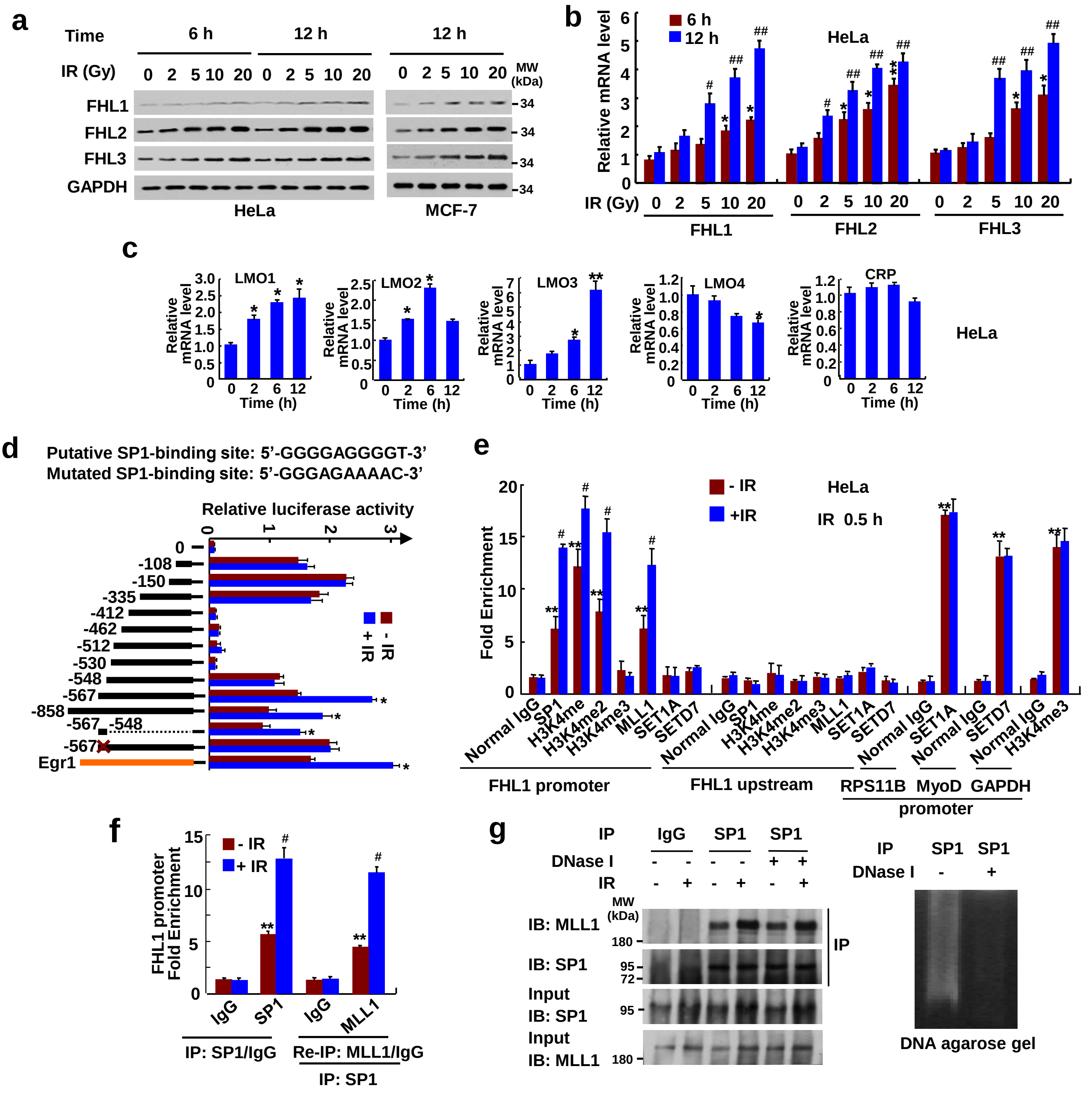

Supplementary Figure 8. SP1 and MLL1 dependent LIM protein induction by IR. (**a**) Immunoblot analysis of HeLa or MCF-7 cells exposed to IR at the indicated doses and harvested at the indicated times. (**b**) Real-time RT-PCR analysis of HeLa cells exposed to IR at the indicated doses and harvested at the indicated times. Data shown are mean ± SD of triplicate measurements that have been repeated 3 times with similar results. $*P < 0.05$, $**P < 0.01$ versus no IR at 6 h. $^{\#}p < 0.05$, $^{\#\#}p < 0.01$ versus no IR at 12 h. (**c**) Real-time RT-PCR analysis of HeLa cells exposed to 10-Gy IR and harvested at the indicated times. Data shown are mean ± SD of triplicate measurements that have been repeated 3 times with similar results. $*P < 0.05$, $**P < 0.01$ versus respective IR at 0 h. (**d**) Luciferase activity of different FHL1 promoter constructs in HeLa cells exposed to 10-Gy IR. The "X" shows the mutated SP1-binding site. Data shown are mean ± SD of triplicate measurements that have been repeated 3 times with similar results. $*P < 0.05$ versus respective construct without IR. (**e**) ChIP analysis of the occupancy of SP1, MLL1 and H3K4 methylation on the FHL1 promoter in HeLa cells. IgG, normal serum. (**f**) Re-ChIP analysis of the occupancy of SP1 and MLL1 on the FHL1 promoter in HeLa cells. Data shown are mean ± SD of triplicate measurements that have been repeated 3 times with similar results (e,f). $**p < 0.01$ versus normal IgG without IR. $^{\#}p < 0.05$ versus respective specific antibody without IR. (**g**) Co-IP analysis of HeLa cells exposed to 10-Gy IR and treated with DNase I. DNA agarose gel electrophoresis showed that DNase I could digest DNA. The *P* values were generated using two-tailed Student's *t*-test (b, c, d, f).

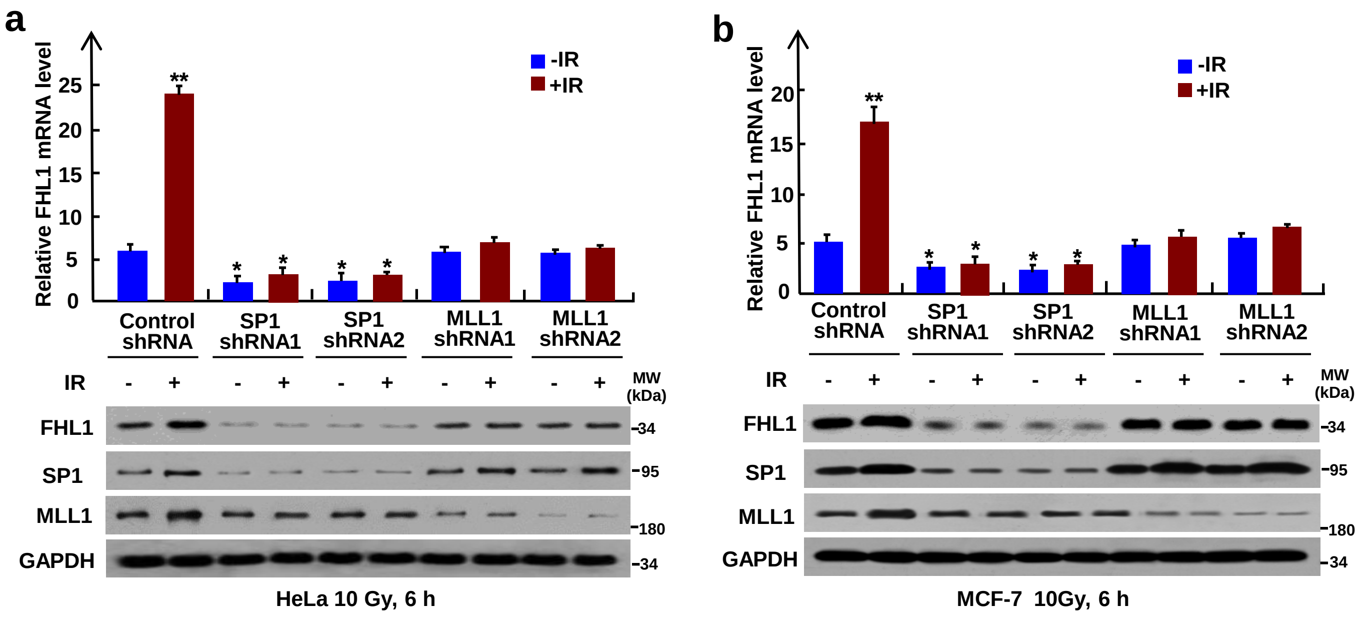

Supplementary Figure 9. Effects of SP1 or MLL1 knockdown on FHL1 expression. HeLa (a) or MCF-7 (b) cells stably infected with SP1 shRNA1/2 or MLL1 shRNA1/2 and exposed to 10-Gy IR. Representative real-time RT-PCR and immunoblot analyses were performed. Data shown are mean ± SD of triplicate measurements that have been repeated 3 times with similar results. The *P* values were generated using two-tailed Student's *t*-test. **P* < 0.05, ***P* < 0.01 versus control shRNA without IR.

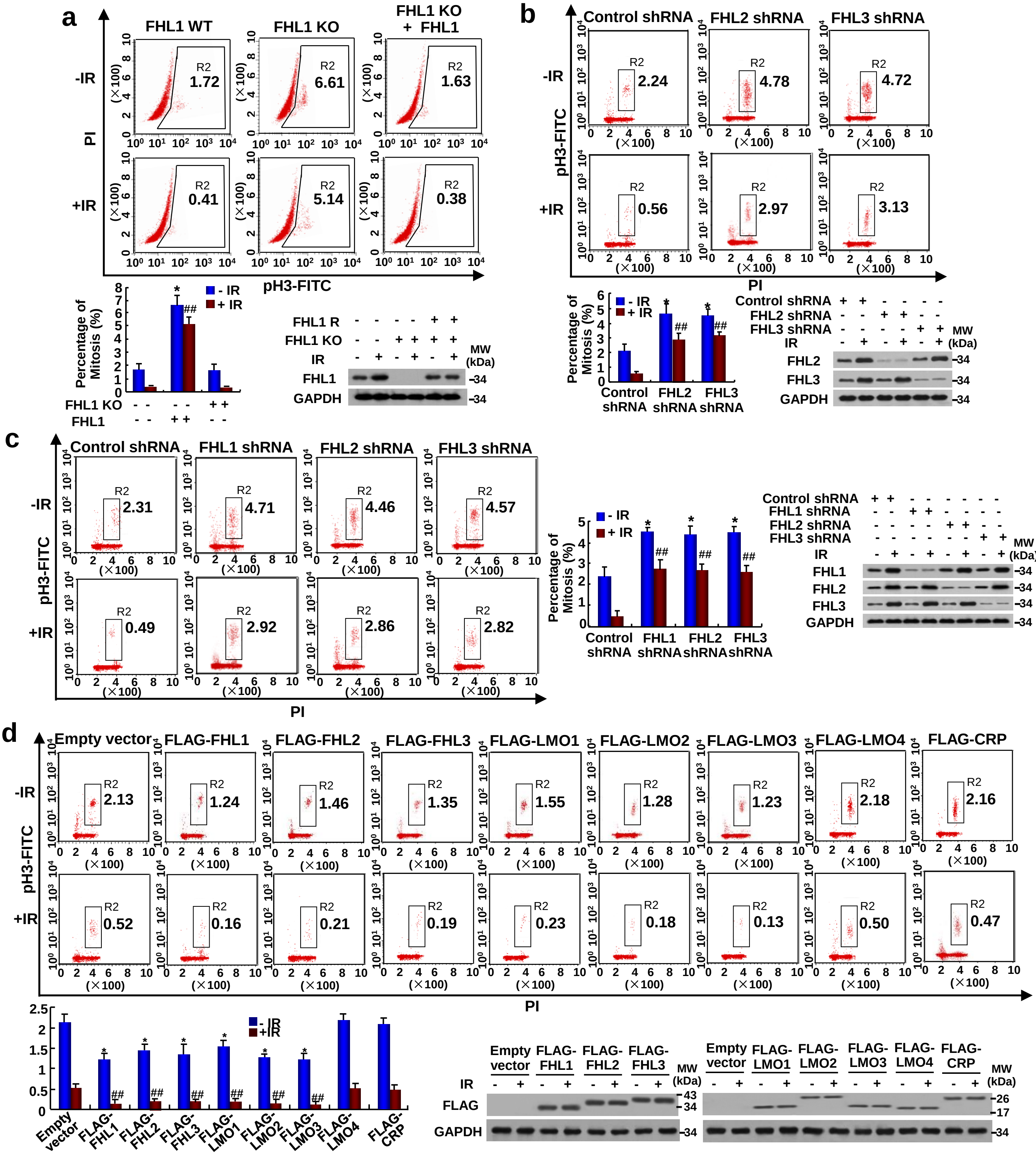

Supplementary Figure 10. LIM proteins regulate the G2/M checkpoint. (**a**) Representative FACS analysis of the proportion of mitotic cells of FHL1 WT or KO MEFs or FHL1 KO MEFs infected with lentivirus harboring FHL1. Immunoblot with the indicated antibodies are also shown. (**b**) Representative FACS analysis of the proportion of mitotic cells in HeLa cells stably infected with FHL2 shRNA or FHL3 shRNA. Representative immunoblot showed knockdown effects of FHL2 shRNA and FHL3 shRNA. (**c**) Representative FACS analysis of the proportion of mitotic cells in MCF-7 cells stably infected with FHL1 shRNA, FHL2 shRNA or FHL3 shRNA. Representative immunoblot showed knockdown effects of these shRNAs. Data shown are mean ± SD of three independent experiments. *p < 0.05 versus control shRNA without IR, $^{\#\#}p$ < 0.01 versus control shRNA with IR (a, b). (**d**) Representative FACS analysis of the proportion of mitotic cells in HeLa cells transiently transfected with FLAG-tagged FHL1-3, LMO1-4 and CRP. Representative immunoblot showed the expression of FLAG-tagged FHL1-3, LMO1-4 and CRP. Data shown are mean ± SD of three independent experiments. *p < 0.05 versus empty vector without IR, $^{\#\#}p$ < 0.01 versus empty vector with IR. The P values were generated using two-tailed Student's t-test (a-d).

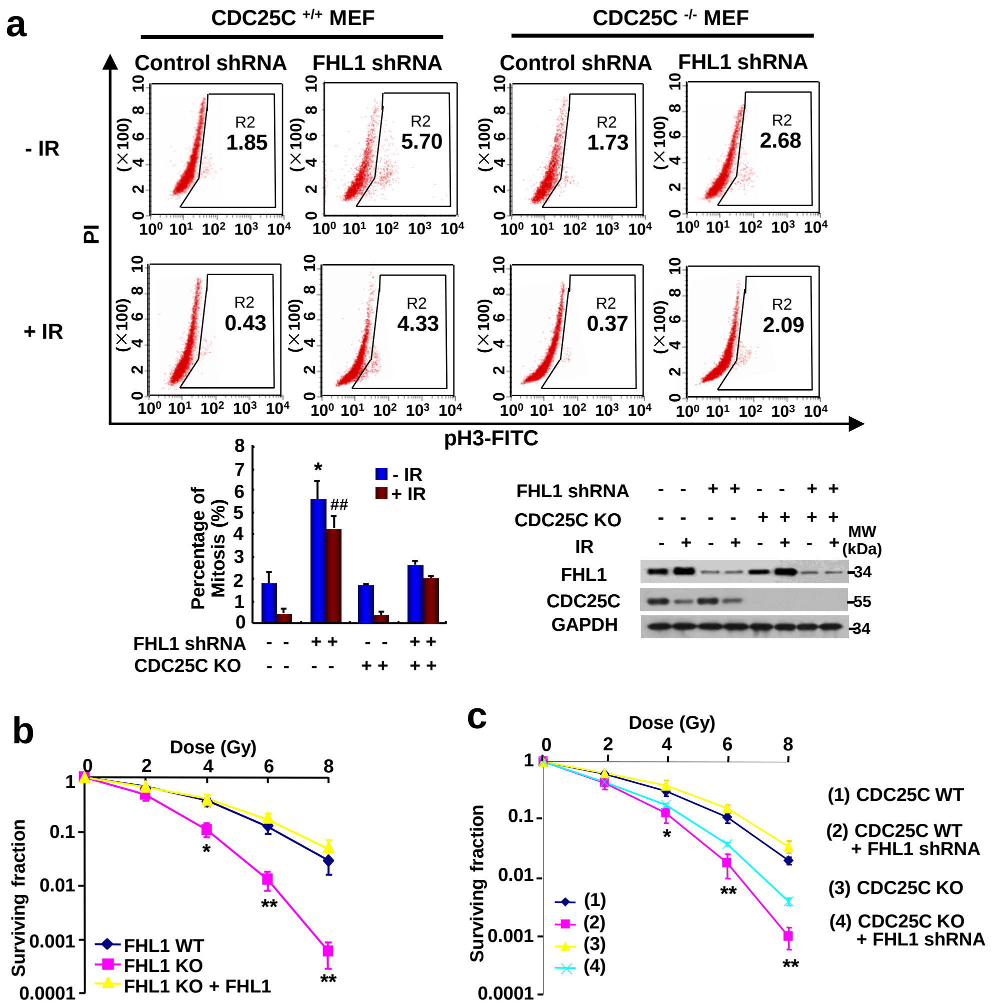

Supplementary Figure 11. FHL1 regulates the G2 checkpoint and radioresistance though CDC25C. (**a**) CDC25C WT or KO MEFs were stably infected with FHL1 shRNA or control shRNA, irradiated and analyzed as in Supplementary Fig. S10a. Data shown are mean ± SD of three independent experiments. **p* < 0.05 versus control shRNA without IR, #*p* < 0.05, ##*p* < 0.01 versus control shRNA with IR (a, b). (**b**) Clonogenic survival assays of FHL1 WT or KO MEFs or FHL1 KO MEFs infected with lentivirus carrying FHL1 and irradiated at the indicated doses. **(c)** Clonogenic survival assays of CDC25C WT or KO MEFs infected with lentivirus harboring FHL1 shRNA or control shRNA, and irradiated at the indicated doses. Data shown are mean ± SD of three independent experiments. **p* < 0.05, ***p* < 0.01 versus corresponding control shRNA (b,c). The *P* values were generated using two-tailed Student's *t*-test (a-c).

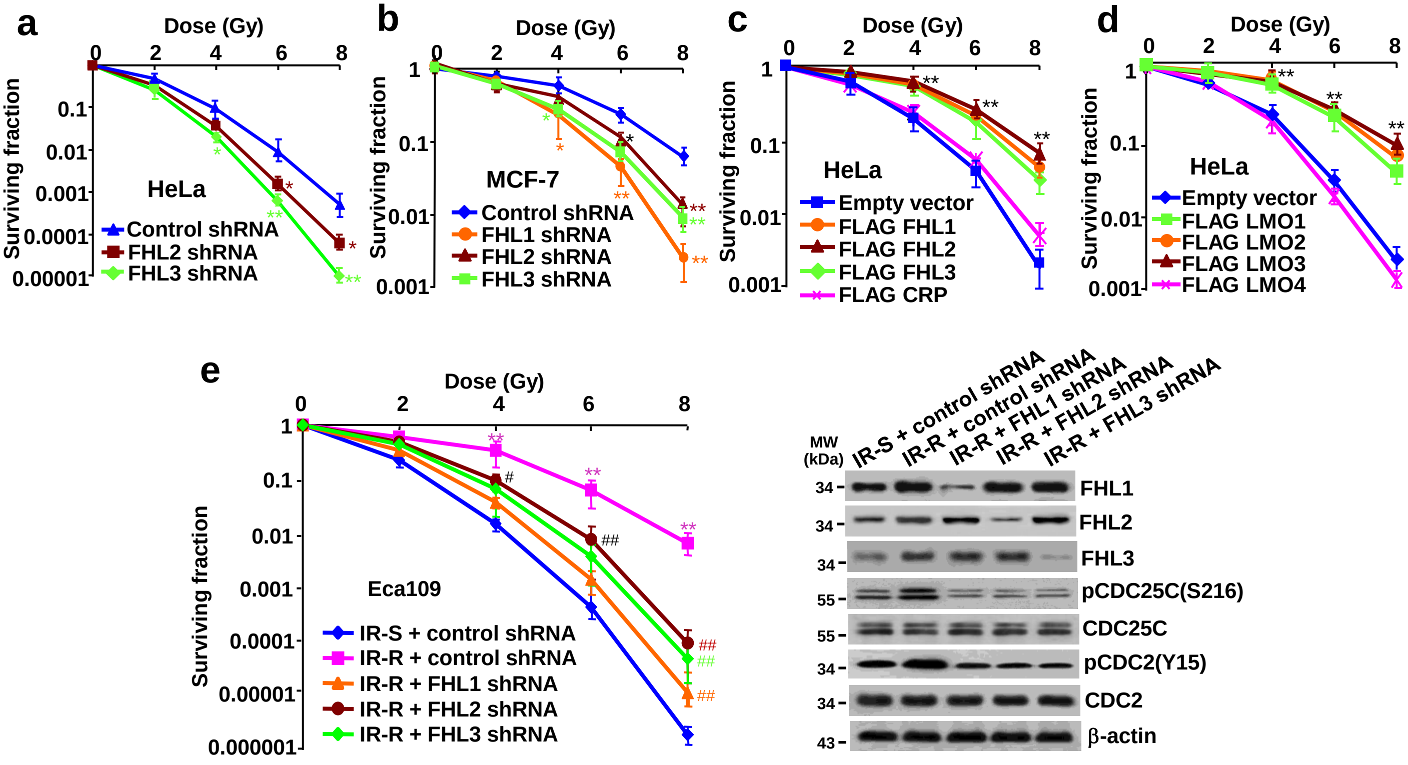

Supplementary Figure 12. LIM proteins modulate cancer cell survival in response to IR. (**a,b**) Clonogenic survival assays of HeLa (a) or MCF-7 (b) cells stably infected with the indicated shRNA constructs and exposed to IR at the indicated doses. Data shown are mean ± SD of three independent experiments. **p* < 0.05, ***p* < 0.01 versus corresponding control shRNA (a,b). (**c,d**) Clonogenic survival assays of HeLa cells transfected with FLAG-tagged FHL1-3, LMO1-4 and CRP, and exposed to IR at the indicated doses. Data shown are mean ± SD of three independent experiments. **p* < 0.05, ***p* < 0.01 versus corresponding empty vector (c,d). (**e**) Clonogenic survival assays of radioresistant Eca109 cells (IR-R) infected with FHL1 shRNA, FHL2 shRNA or FHL3 shRNA, and exposed to IR at the indicated doses. The parental Eca 109 cell line (IR-S) was used as a control. Representative immunoblot revealed the expression of FHL1-3 and G2/M-related proteins. Data shown are mean ± SD of three independent experiments. **p* < 0.05, ***p* < 0.01 versus control shRNA in IR-S cells. $^{\#}p < 0.05$, $^{\#\#}p < 0.01$ versus control shRNA in IR-R cells. The *P* values were generated using two-tailed Student's *t*-test (a-e).

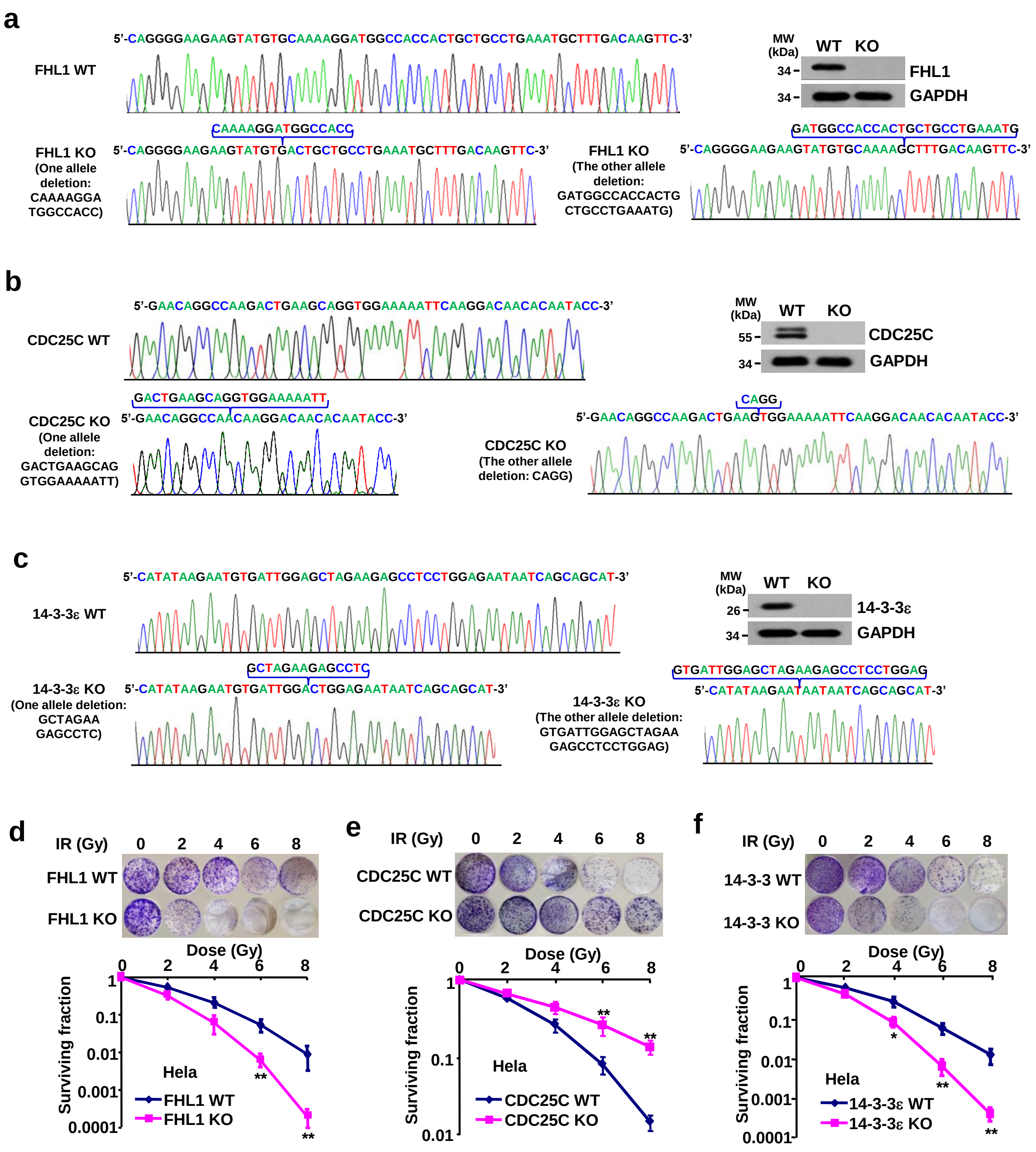

Supplementary Figure 13. The effect of radiation on the survival of FHL1, CDC25C or 14-3-3ε knockout Hela cells. (a-c) Identification of FHL1, CDC25C or 14-3-3ε knockout Hela cell lines by DNA sequencing and immunoblot with the indicated antibodies. **(d-f)** FHL1, 14-3-3ε or CDC25C KO Hela cell lines and their corresponding WT cell lines were irradiated at the indicated doses and analyzed for clonogenic survival assays. Data shown are mean ± SD of three independent experiments. The *P* values were generated using two-tailed Student's *t*-test. *$p < 0.05$, **$p < 0.01$ versus WT cells.

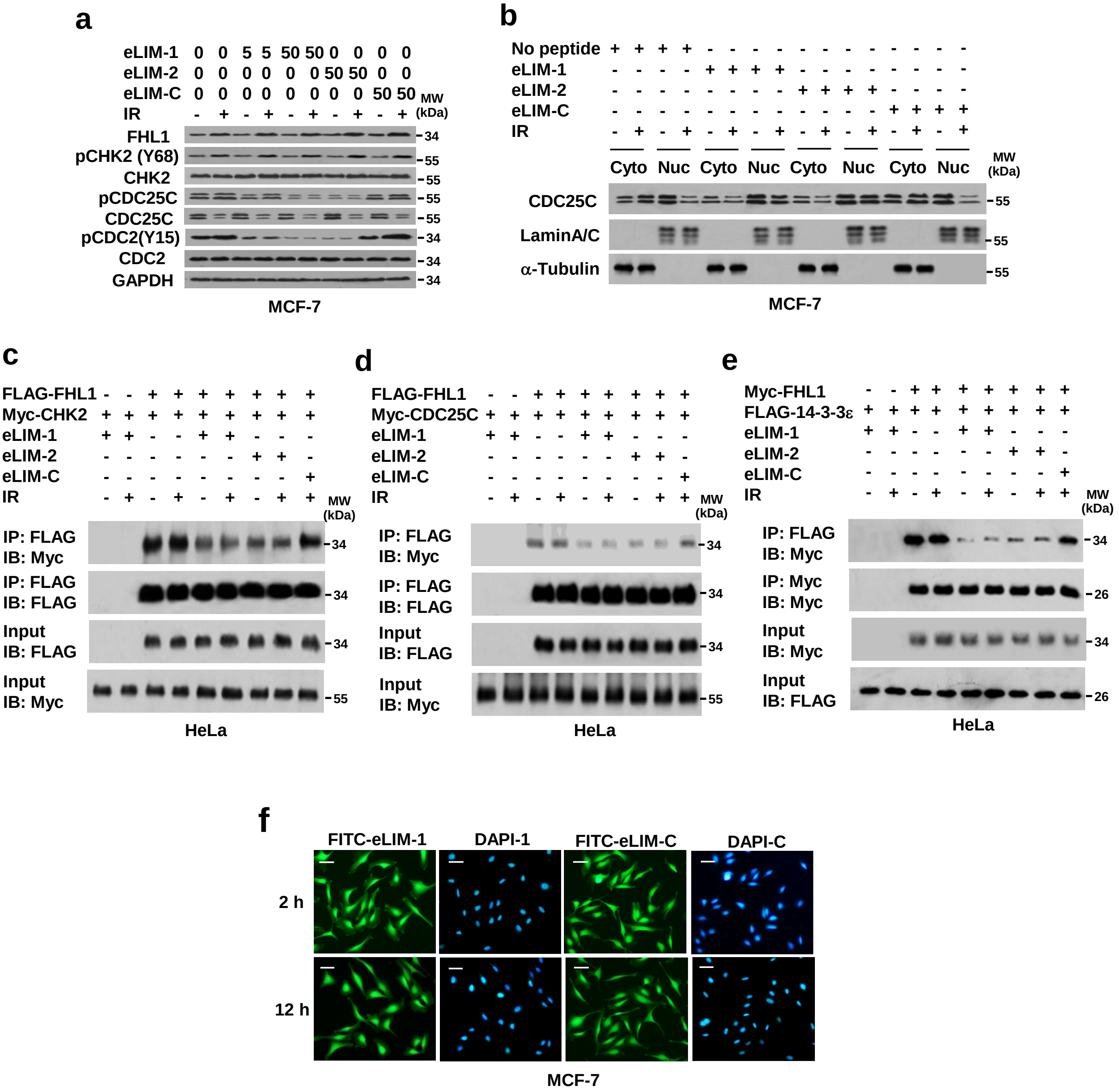

Supplementary Figure 14. eLIM motif promotes CDC25C phosphorylation and nuclear CDC25C. (**a**) Immunoblot analysis of MCF-7 cells treated with 50 nM eLIM peptides and exposed to 8-Gy IR. (**b**) Immunoblot analysis of subcellular localization of CDC25C in MCF-7 cells treated with 50 nM eLIM peptides and exposed to 8-Gy IR. (**c-e**) Co-IP analysis of HeLa cells transfected with the indicated plasmids, treated with 50 nM eLIM peptides and exposed to 8-Gy IR. (**f**) MCF-7 cells were treated with the indicated peptides labeled with FITC (50 nM) at the indicated times. The nuclei were stained with DAPI (blue). The location of the peptides (green) was visualized by a fluorescent microscope. Scale bar: 100 μm.

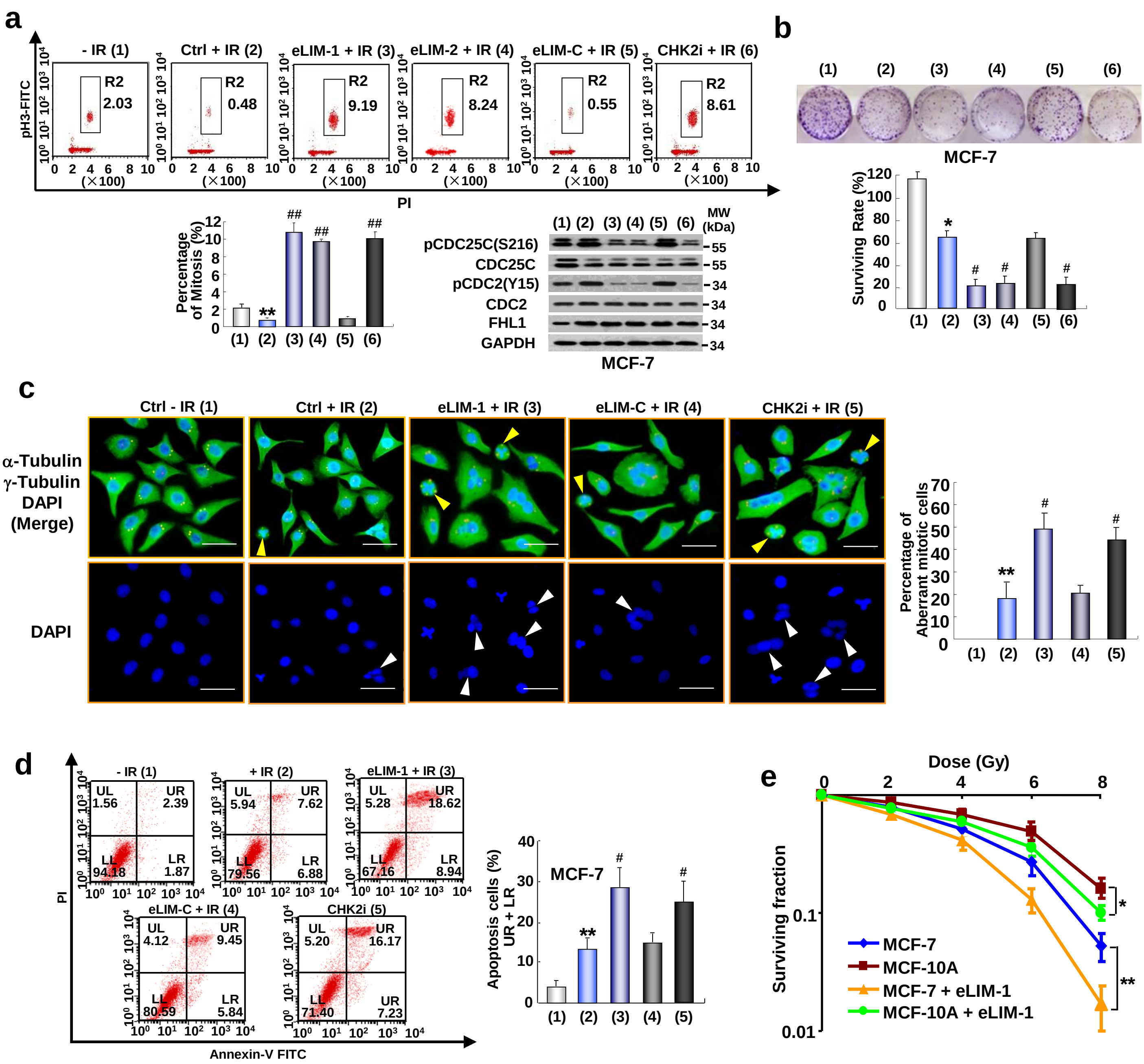

Supplementary Figure 15. eLIM increases cancer cell radiosensitivity via mitotic catastrophe and apoptosis. (**a**) Representative FACS analysis of the proportion of mitotic cells in MCF-7 cells treated with the indicated peptides (50 nM) or CHK2 inhibitor II (100 nM). Representative immunoblot with the indicated antibodies is shown. (**b**) Clonogenic survival assays of MCF-7 cells treated as in (a). (**c**) MCF-7 cells treated as in (a) were stained with anti-α-Tubulin (green) and anti-γ-tubulin (red). The nuclei were stained with DAPI (blue). Yellow arrows indicate cells with multipolar spindles and white arrows indicate cells with polymorphologic nucleus. The percentages of aberrant mitotic cells were plotted. Scale bar: 100 μm. (**d**) Representative FACS analysis of apoptosis stained with Annexin V and PI in MCF-7 cells treated as in (a). Data shown are mean ± SD of three independent experiments. **p* < 0.05, ***p* < 0.01 versus no peptide without IR, #*p* < 0.05, ##*p* < 0.01 versus no peptide with IR (a-d). (**e**) Clonogenic survival assays of MCF-7 or MCF-10A cells treated with or without 50 nM eLIM-1. Data shown are mean ± SD of three independent experiments. (**p* < 0.05, ***p* < 0.01). The *P* values were generated using two-tailed Student's *t*-test (d, e).

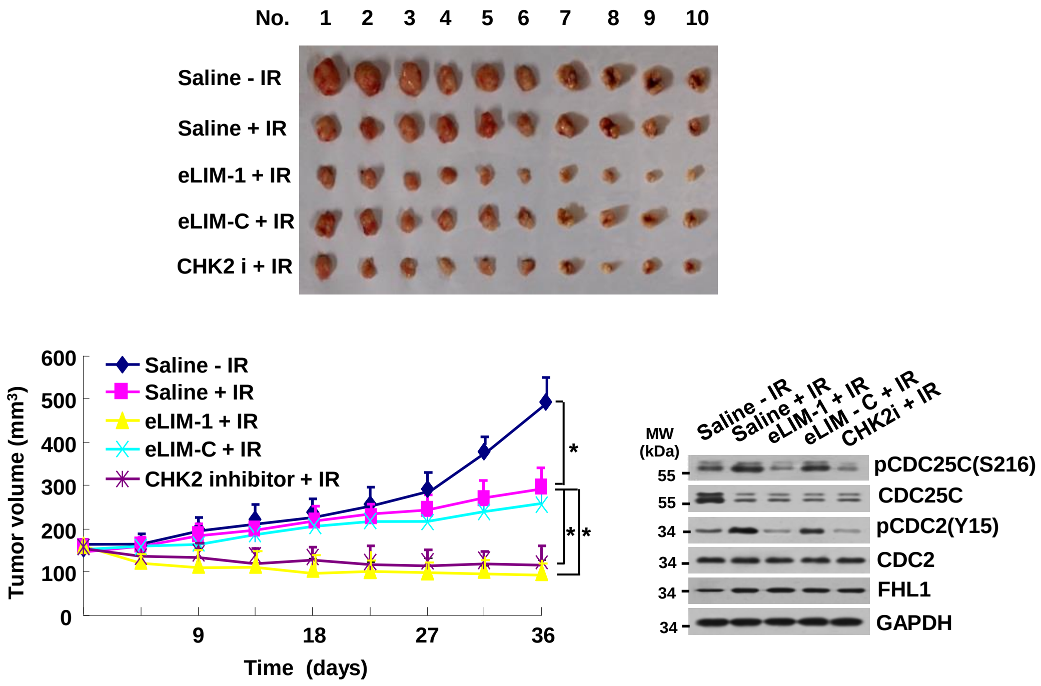

Supplementary Figure 16. eLIM-1 peptide increases tumor radiosensitivity. Volume of xenograft tumors derived from MCF-7 cells treated with the indicated peptide or CHK2i as indicated. The different number (1 to 10) refers to different mice. Data are shown as mean ± SD (n = 10). The *P* values were generated using two-tailed Student's *t*-test. (*p < 0.05, **p < 0.01 at 36 days). Representative tumor tissues (No. 5) were subjected to immunoblot analysis with the indicated antibodies.

Fig.1b

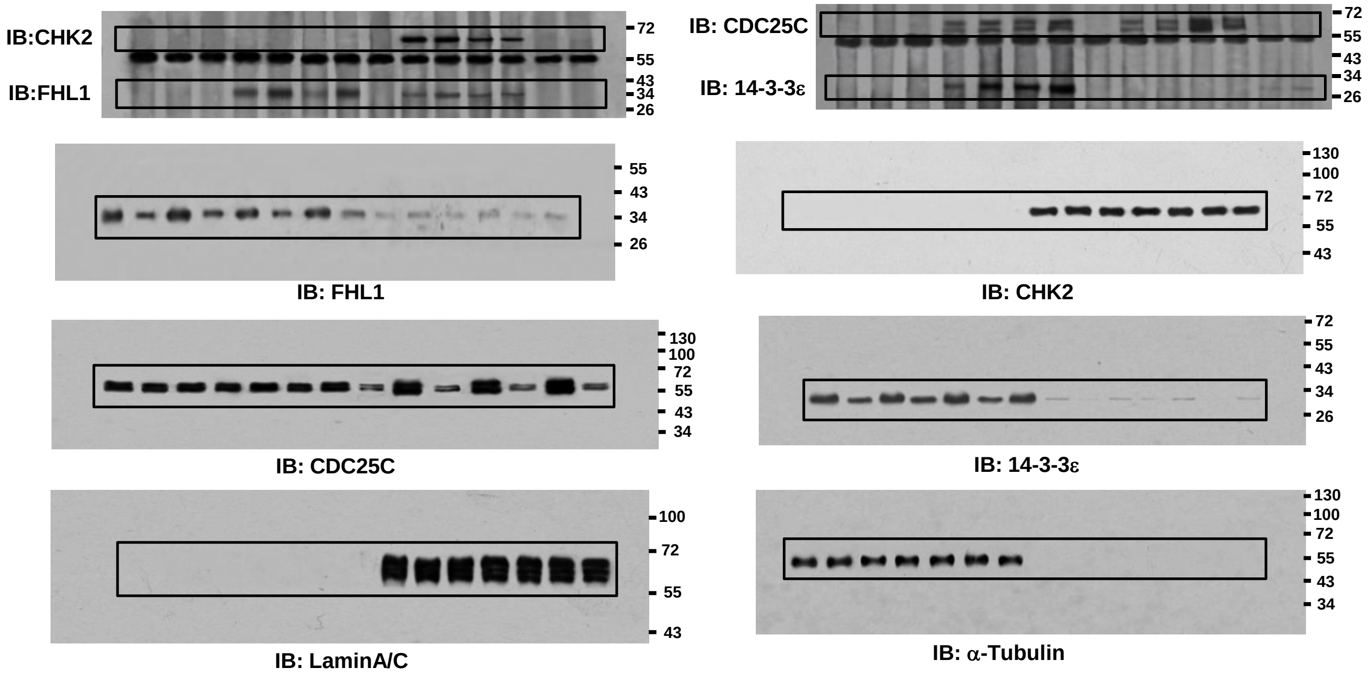

Fig.1c

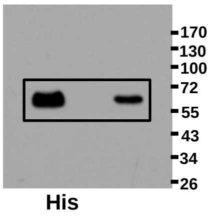

Fig.1d

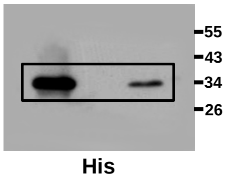

Fig.1e

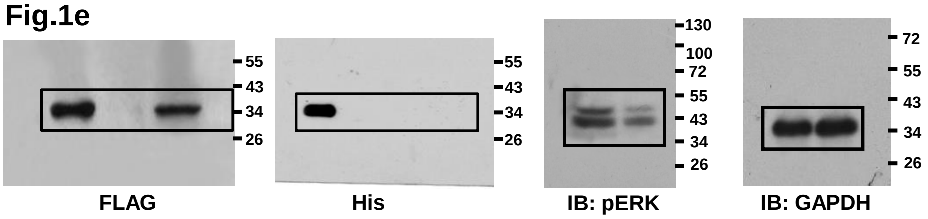

Fig.1g

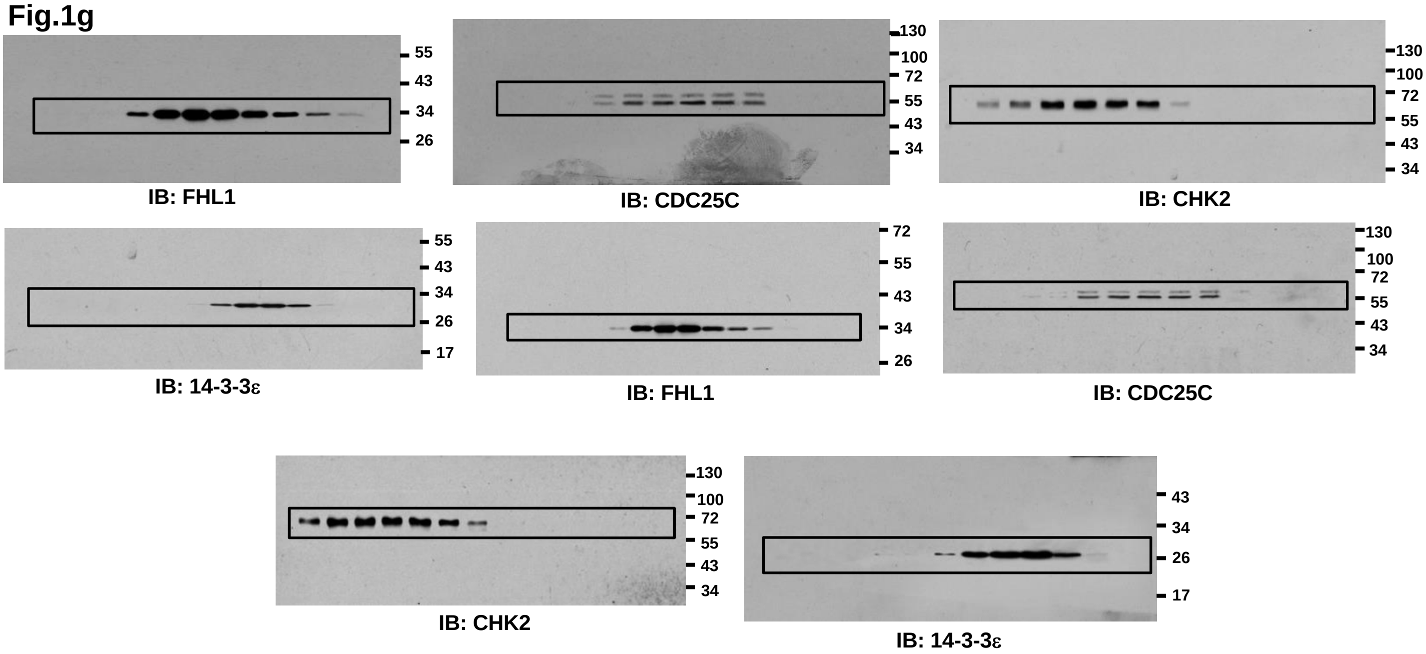

Supplementary Figure 17 (continued below)

Fig.2a

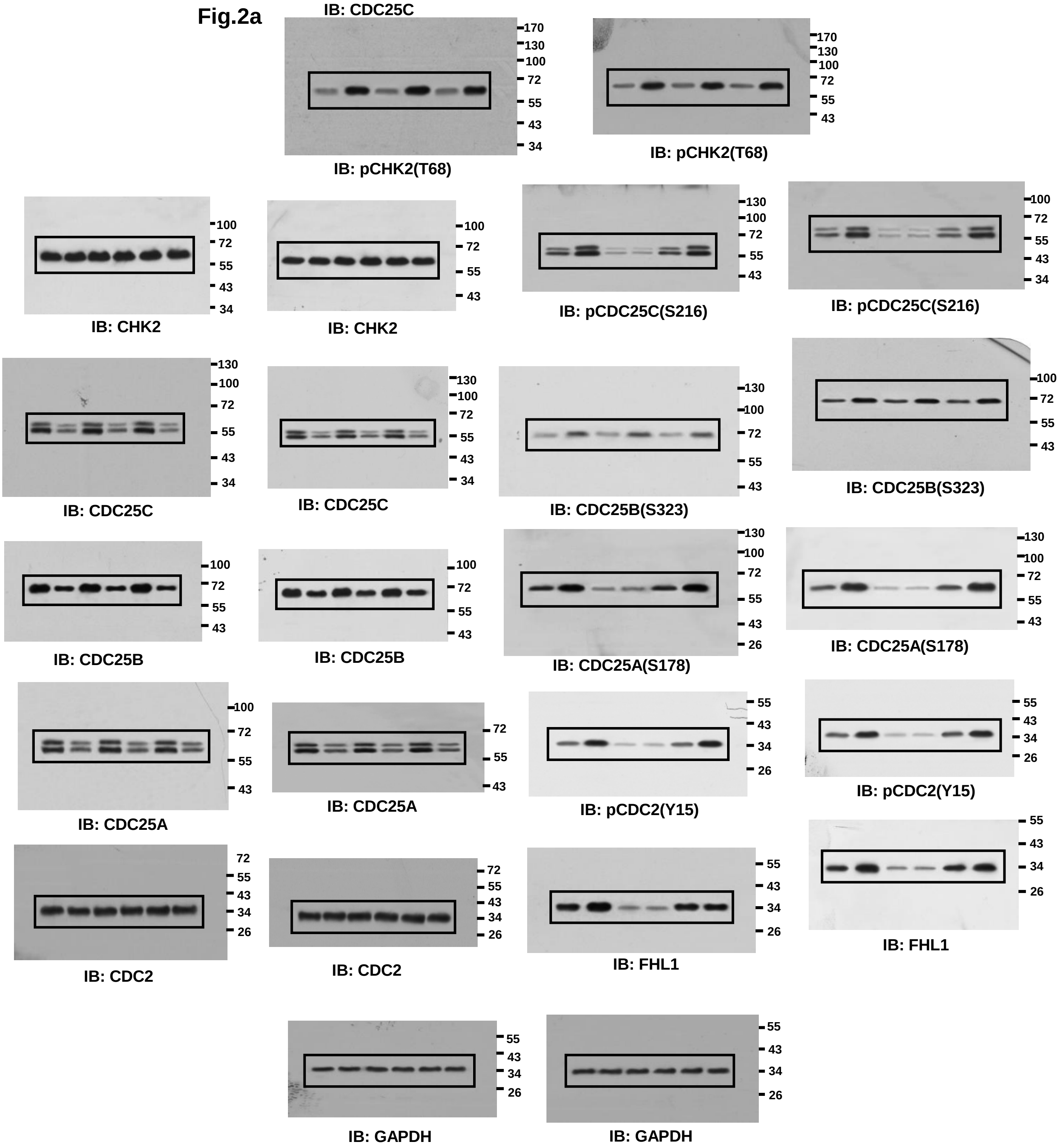

Supplementary Figure 17 (continued below)

Fig.2b

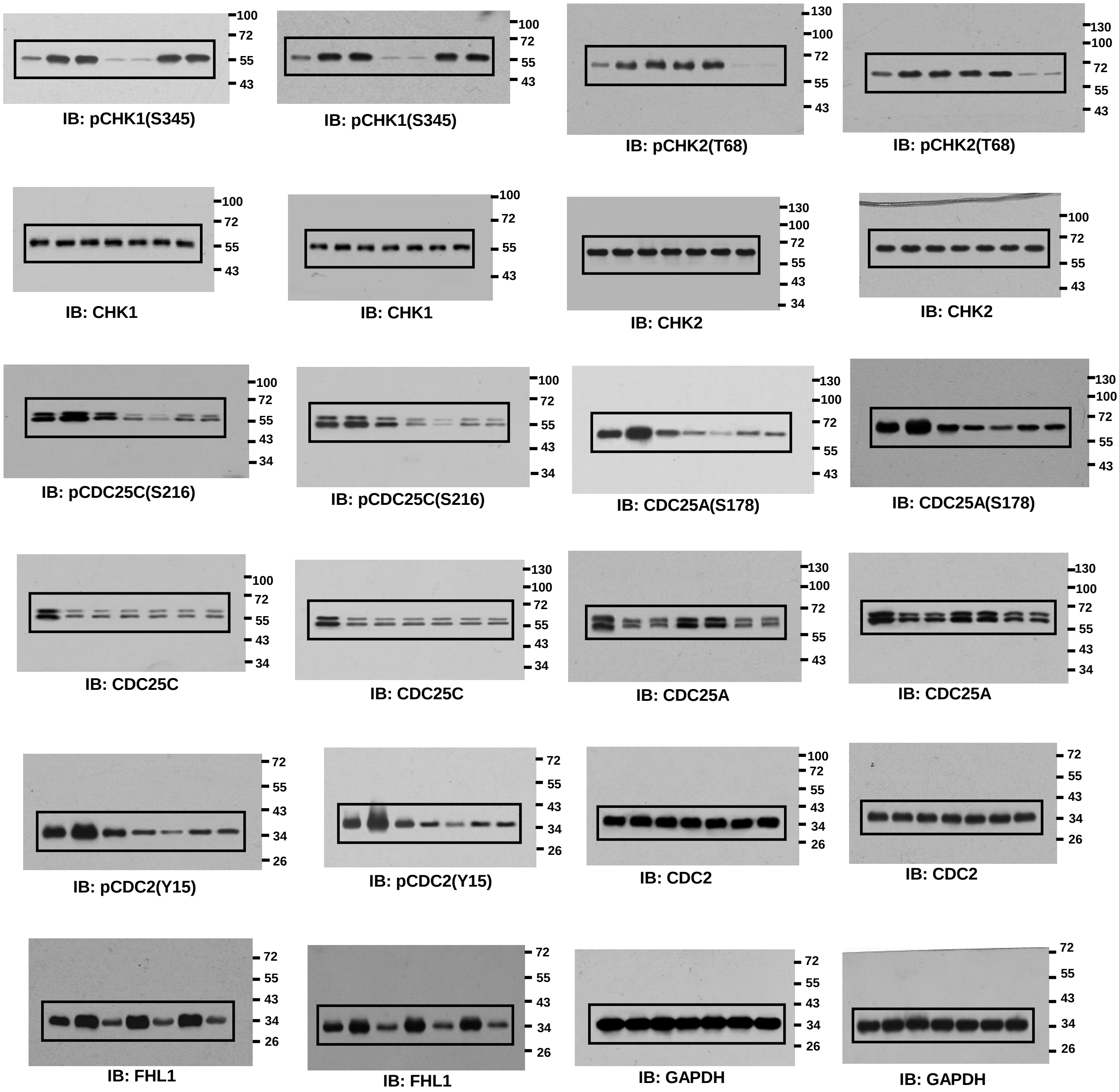

Supplementary Figure 17 (continued below)

Fig.2c

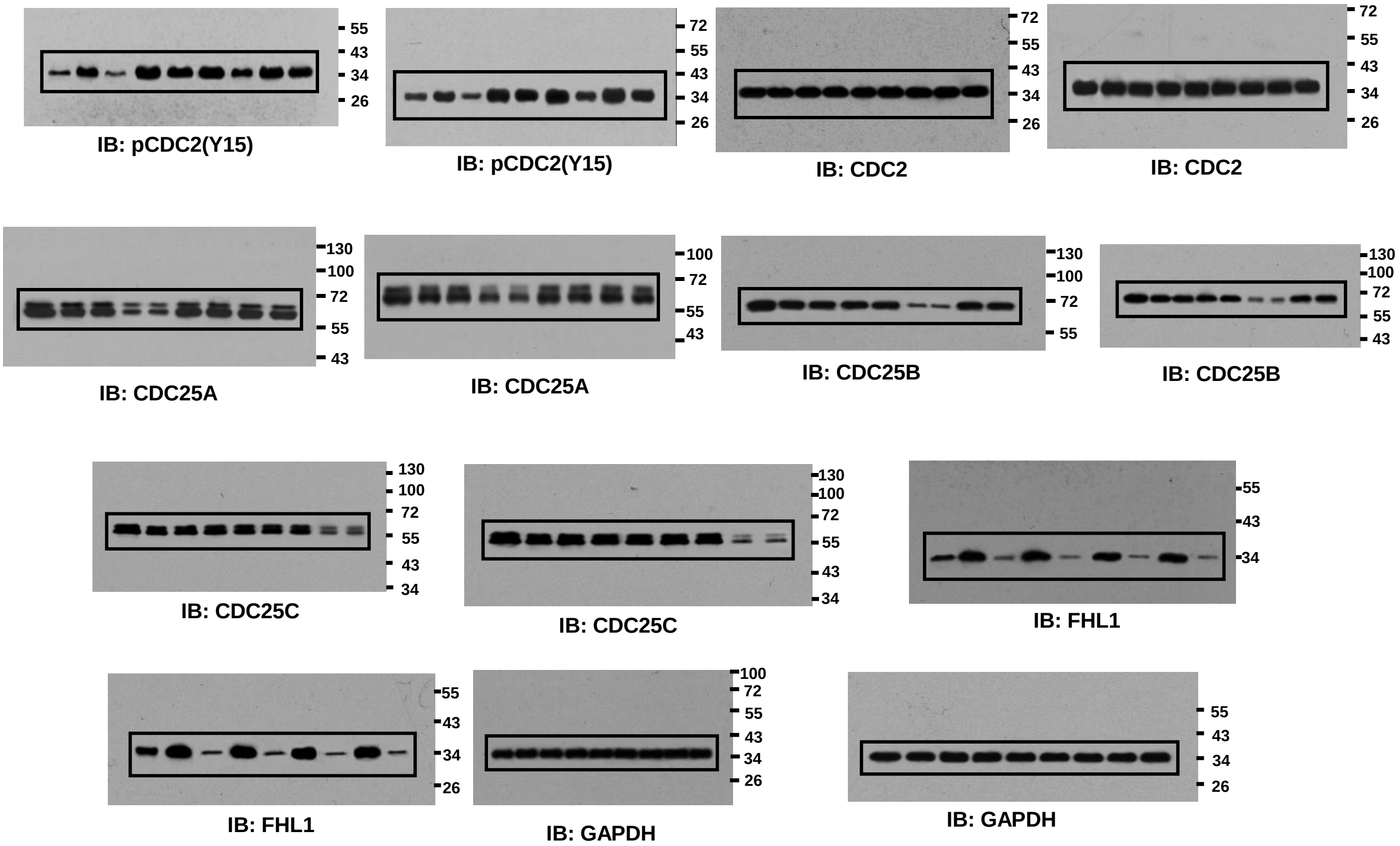

Supplementary Figure 17 (continued below)

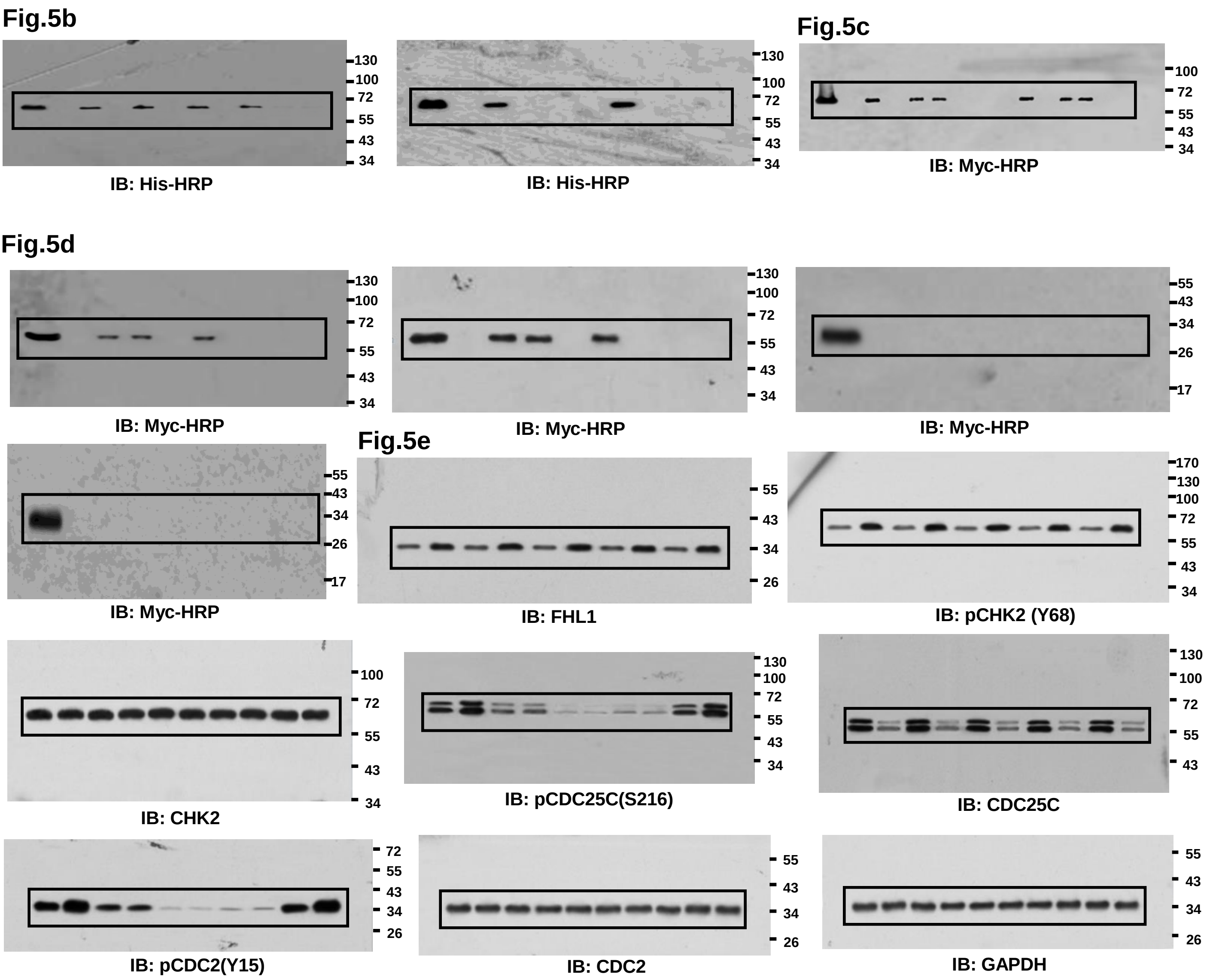

Supplementary Figure 17. Uncropped images of the most important blots

Factors	Univariate			Multivariate		
	p	**HR**	**95% CI**	***p***	**HR**	**95% CI**
**Disease-free survival**						
Age：≤ 50 vs >50 years	0.329	0.984	0.951-1.017	0.872	0.997	0.964-1.030
Tumor size: ≤20 vs >20 ≤50 vs >50 mm	8.249×10^{-6}	3.815	2.118-6.872	0.002	2.697	1.459-4.986
Nodal status: N0 vs N1 vs N2 vs N3	1.385×10^{-5}	4.078	2.164-7.687	0.002	2.893	1.497-5.590
Grade: I vs II vs III	0.007	2.253	1.244-4.080	0.231	1.790	0.979-3.274
ERα: negative vs positive	0.012	0.471	0.262-0.847	0.852	0.775	0.661-1.252
PR: negative vs positive	0.332	0.749	0.417-1.344	0.819	0.833	0.738-1.588
HER2: negative vs positive	0.001	2.964	1.570-5.596	0.009	2.361	1.238-4.506
FHL1: low expression vs high expression	4.605×10^{-4}	2.946	1.610-5.393	0.001	2.836	1.540-5.223
**Overall survival**						
Age：≤ 50 vs >50 years	0.506	0.988	0.953-1.024	0.916	1.007	0.519-1.323
Tumor size: ≤20 vs >20 ≤50 vs >50 mm	1.379×10^{-5}	3.902	2.112-7.209	0.005	2.494	1.321-4.709
Nodal status: N0 vs N1 vs N2 vs N3	6.921×10^{-7}	5.800	2.897-11.612	2.064×10^{-4}	3.969	1.916-8.219
Grade: I vs II vs III	0.003	2.501	1.354-4.621	0.022	2.109	1.115-3.991
ERα: negative vs positive	0.001	0.357	0.192-0.666	0.142	0.672	0.502-1.131
PR: negative vs positive	0.140	0.633	0.345-1.161	0.306	0.781	0.424-0.915
HER2: negative vs positive	0.001	2.856	1.505-5.421	0.276	2.212	0.784-2.872
FHL1: low expression vs high expression	8.874×10^{-5}	3.756	1.938-7.280	4.787×10^{-4}	3.295	1.687-6.437

Supplementary Table 1. Cox univariate and multivariate analysis of disease-free survival and overall survival in breast cancer patients treated with radiotherapy.

Factors	Univariate			Multivariate		
	p	**HR**	**95% CI**	***p***	**HR**	**95% CI**
**Disease-free survival**						
Age：≤ 50 vs >50 years	0.962	0.999	0.968-1.031	0.181	1.086	0.887-1.197
Tumor size: < 4 vs ≥4 cm	0.009	2.435	1.244-4.766	0.003	2.904	1.457-5.827
FIGO stage: Ⅰ/Ⅱ vs Ⅲ/Ⅳ	0.007	2.494	1.287-4.835	0.019	2.302	1.149-4.609
SCC-Ag: < 1.5 ng/mL vs ≥1.5 ng/mL	0.025	1.776	0.337-1.905	0.235	1.987	0.623-2.674
FHL1: low expression vs high expression	0.007	2.671	1.302-5.478	0.066	2.354	1.110-4.991

Supplementary Table 2. Cox univariate and multivariate analysis of disease-free survival in cervical cancer patients treated with radiotherapy.

Factors	Univariate			Multivariate		
	p	HR	95% CI	*p*	HR	95% CI
**Disease-free survival**						
Age：≤ 50 vs >50 years	0.623	0.990	0.949-1.032	0.631	1.006	0.833-1.027
Tumor size: ≤20 vs >20 ≤50 vs >50 mm	0.001	6.519	2.134-19.914	0.002	7.104	2.004-25.184
Nodal status: N0 vs N1 vs N2 vs N3	0.001	5.773	2.040-16.336	0.035	3.342	1.088-10.266
Grade: I vs Ⅱ vs III	2.336×10^{-4}	3.640	1.829-7.244	0.002	3.275	1.535-6.987
ERα: negative vs positive	0.013	0.271	0.097-0.761	0.030	0.294	0.097-0.886
PR: negative vs positive	0.281	0.583	0.219-1.554	0.952	0.675	0.297-1.376
HER2: negative vs positive	0.003	4.288	1.658-11.089	0.020	3.451	1.218-9.779
FHL1: low expression vs high expression	0.321	0.619	0.240-1.597	0.071	0.882	0.440-1.080
**Overall survival**						
Age：≤ 50 vs >50 years	0.414	0.983	0.945-1.056	0.783	0.992	0.360-1.846
Tumor size: ≤20 vs >20 ≤50 vs >50 mm	0.007	4.662	1.530-14.203	0.375	3.965	1.732-13.557
Nodal status: N0 vs N1 vs N2 vs N3	2.973×10^{-4}	6.991	2.438-20.048	3.522×10^{-4}	8.306	2.601-26.526
Grade: I vs Ⅱ vs III	0.001	3.307	1.683-6.495	0.015	2.580	1.206-5.517
ERα: negative vs positive	0.006	0.238	0.085-0.692	0.046	0.327	0.109-0.979
PR: negative vs positive	0.108	0.447	0.168-1.192	0.658	0.392	0.213-0.969
HER2: negative vs positive	0.003	4.055	1.627-10.105	0.050	2.662	1.001-7.080
FHL1: low expression vs high expression	0.310	0.622	0.248-1.556	0.783	0.703	0.445-1.628

Supplementary Table 3. Cox univariate and multivariate analysis of disease-free survival and overall survival in breast cancer patients without radiotherapy.

Factors	Univariate			Multivariate		
	p	**HR**	**95% CI**	***p***	**HR**	**95% CI**
**Disease-free survival**						
Age：≤ 50 vs >50 years	0.588	0.938	0.928-1.043	0.900	0.995	0.917-1.079
Tumor size: < 4 vs ≥4 cm	0.147	2.348	0.740-7.451	0.248	2.320	1.418-6.204
FIGO stage: Ⅰ/Ⅱ vs Ⅲ/Ⅳ	0.063	3.354	0.936-12.022	0.125	3.876	1.225-15.588
SCC-Ag: < 1.5 ng/mL vs ≥1.5 ng/mL	0.292	1.537	0.169-1.705	0.913	1.109	0.175-7.011
FHL1: low expression vs high expression	0.769	1.193	0.366-3.886	0.412	4.635	0.700-10.703

Supplementary Table 4. Cox univariate and multivariate analysis of disease-free survival in cervical cancer patients without radiotherapy.

**Gene name**	**NCBI Reference Sequence number**
FHL1	NM_001449.4
FHL2	NM_001450.3
FHL3	NM_004468.4
LMO1	NM_002315.2
LMO2	NM_005574.3
LMO3	NM_018640.4
LMO4	NM_006769.3
CRP	NM_004078.2
14-3-3ε	NM_006761.4
14-3-3σ	NM_006142.3
14-3-3ζ	NM_003406.3
CDC25A	NM_001789.2
CDC25B	NM_021873.3
CDC25C	NM_001790.4
SP1	NM_138473.2
MLL1	NM_001197104.1

Supplementary Table 5. The number of the NCBI Reference Sequence for genes used in this study.

Gene	Target sequence (5’→3’)
FHL1 [1]	AAGGAGGTGCACTATAAGAAC
FHL2 [1]	AACTGCTTCTGTGACTTGTAT
FHL3 [1]	AAGTACATCCAGACAGACAGC
14-3-3ε-1 [2]	AAACCATTACAACGAAGTCCCTCCC
14-3-3ε-2 [2]	ACCAACGCATCCTATTCGC
CDC25A-1 [3]	AAGGAAAATGAAGCCTTTGAG
CDC25A-2 [4]	CCGATTCAGGTTTCTGTCT
CDC25B-1 [5]	GCCGGATCATTCGAAACGA
CDC25B-2 [6]	TCCTCCCTGTCGTCTGAAT
CDC25C-1 [7]	AACCCCAAAATGTTGCCTCGA
CDC25C-2 [8]	AAAAATGTTGCCTCGATCTTTC
SP1-1 [9]	GGTAGCTCTAAGTTTTGAT
SP1-2 [9]	GGTCATTTCTTTGCTTATG
MLL-1[10]	GGACAAGAGTAGAGAGAGA
MLL-2[10]	AGGAGAAGGAAGAGGCAAA

Supplementary Table 6. The cDNA target sequences of shRNAs or siRNAs.

Gene	Forward (5'→3')	Reverse (5'→3')
FHL1	CGCTGTGGAGGACCAGTATTAC	TAGTCGTGCCAGGATTGTCCTT
FHL2	GCACAAGGAGTGCTTCGTGTG	GTCCGCTGATGGGGTTGGTG
FHL3	TCCCGGGATGAAGATCCCTAC	GTTGTGGTGCCAGTGTCGGTC
LMO1	GCAGCAAGCTGATCCCAGCC	TTACTGAACTTGGGATTCAAAGG
LMO2	CAAGCGGATTCGTGCCTATGAG	CTATATCATCCCATTGATCT TAGT
LMO3	GTTTACCACCTGGACTGCTTTG	TCAGCGAACCTGGGGTGCAT
LMO4	GCGGTGCTTGCAGCGCTTGC	CCATTGATGTAGTGAAACCG
CRP	ACCACCAACCCCAATGCAT	AGAAGATCGGCGGCTCTGAG
β-actin	ATCACCATTGGCAATGAGCG	TTGAAGGTAGTTTCGTGGAT

Supplementary Table 7. Primers used for real-time RT-PCR.

Supplementary References

[1] Ding, L.H. *et al.* Human four-and-a-half LIM family members suppress tumor cell growth through a TGF-β-like signaling pathway. *J. Clin. Invest.* **119**, 349-361 (2009).

[2] Liu, T.A. *et al.* 14-3-3ε overexpression contributes to epithelial-mesenchymal transition of hepatocellular carcinoma. *PLoS One* **8**, e57968 (2013).

[3] Mailand, N. *et al.* Regulation of G(2)/M events by Cdc25A through phosphorylation -dependent modulation of its stability. *EMBO. J.* **21**, 5911-5920 (2002).

[4] Zhang, Y. *et al.* The Chk1/Cdc25A pathway as activators of the cell cycle in neuronal death induced by camptothecin. *J. Neurosci.* **26**, 8819-8828 (2006).

[5] Schmidt, A. *et al.* Rho GTPases regulate PRK2/PKN2 to control entry into mitosis and exit from cytokinesis. *EMBO. J.* **26**, 1624-1636 (2007).

[6] Kramer, A. *et al.* Centrosome-associated Chk1 prevents premature activation of cyclin-B-Cdk1 kinase. *Nat. Cell Biol.* **6**, 884-891 (2004).

[7] Goh, W.C., Manel, N. & Emerman, M. The human immunodeficiency virus Vpr protein binds Cdc25C: implications for G2 arrest. *Virology* **318**, 337-349 (2004).

[8] Turowski, P., Franckhauser, C. & Morris, M.C. Functional cdc25C dual-specificity phosphatase is required for S-phase entry in human cells. *Mol. Biol. Cell* **14**, 2984-2998 (2003).

[9] Jungert, K. *et al.* Sp1 is required for transforming growth factor-beta-induced mesenchymal transition and migration in pancreatic cancer cells. *Cancer Res.* **67**, 1563-1570 (2007).

[10] Wang. X. *et al.* MLL1, a H3K4 methyltransferase, regulates the TNFα-stimulated activation of genes downstream of NF-κB. *J. Cell Sci.* **125**, 4058-4066 (2012).
